# Streamlined and efficient patient-specific modeling for lumbar spine segmentation and finite element analysis

**DOI:** 10.1038/s41598-025-19664-6

**Published:** 2025-10-13

**Authors:** Mohsen Ahmadi, Hanxi Chen, Maohua Lin, Debojit Biswas, James Doulgeris, Yufei Tang, Erik D. Engeberg, Javad Hashemi, Gui Pires, Frank D. Vrionis

**Affiliations:** 1 Department of Electrical and Computer Science, Florida Atlantic University, Boca Raton, Florida USA; 2 Department of Biomedical Engineering, Florida Atlantic University, Boca Raton, Florida USA; 3 Department of Ocean and Mechanical Engineering, Florida Atlantic University, Boca Raton, Florida USA; 4 Center for Complex Systems, Florida Atlantic University, Boca Raton, Florida USA; 5 SurGenTec LLC, Boca Raton, Florida USA; 6 Department of Neurosurgery, Marcus Neuroscience Institute, Boca Raton Regional Hospital, Boca Raton, Florida USA

**Keywords:** Automated finite element modeling, Lumbar spine, Segmentation, Cortical and cancellous bone extraction, Ligament attachment automation, Bone, Computational models, Mechanical engineering

## Abstract

Advancing our understanding of spinal biomechanics through Finite Element Analysis (FEA) is essential for clinical decision-making and biomechanical research. Traditional FEA workflows are hindered by manual segmentation and meshing, introducing inconsistencies, user variability, and lengthy processing times. This study presents a streamlined, patient-specific modeling methodology for the lumbar spine that fundamentally transforms the FEA preprocessing pipeline. By integrating deep learning-based segmentation with advanced computational tools such as the GIBBON library and FEBio, our approach minimizes manual intervention, accelerates model preparation, and enhances both accuracy and reproducibility. The proposed workflow enables precise extraction and meshing of key anatomical structures including cortical and cancellous bone, intervertebral discs, ligaments, and cartilage directly from clinical CT imaging data. Robust segmentation techniques ensure accurate identification and separation of these components, which are subsequently converted into high-resolution surface and volumetric meshes. To optimize model fidelity and computational efficiency, the pipeline incorporates geometric smoothing and adaptive mesh decimation. Ligament attachment is addressed through an innovative coordinate-based framework that leverages anatomical landmarks for automated placement and orientation, overcoming a major challenge in FEA preprocessing. The results demonstrate that the resulting subject-specific models reproduce physiological biomechanics with high fidelity. Range of Motion and stress distribution outcomes closely match experimental data and established numerical models, confirming the pipeline’s accuracy. Importantly, preparation time is reduced from days to just hours, delivering an efficient, reproducible workflow. By unifying segmentation, meshing, and ligament modeling in a single efficient framework, this study establishes a scalable platform for rapid, reliable, and anatomically accurate FEA of the lumbar spine, with significant implications for clinical diagnostics and preoperative planning.

## Introduction

The lumbar spine is a fundamental component of the human musculoskeletal system, playing a critical role in supporting body weight, facilitating movement, and protecting the spinal cord. Its biomechanical behavior is determined by the complex interaction of vertebrae, intervertebral discs, ligaments, and cartilage, which together maintain spinal stability and distribute mechanical loads^1–4^. Finite Element Analysis (FEA) has emerged as a powerful computational tool for investigating spinal biomechanics, allowing researchers to analyze load distribution, stress patterns, and range of motion (ROM) under various physiological and pathological conditions^5–7^. However, accurate spinal modeling necessitates precise geometry reconstruction, material property assignment, and appropriate biomechanical representation, which can be challenging due to inter-subject variability in anatomical structures and tissue properties^8–10^. Numerous studies have sought to refine FEA models to better capture the complexities of spinal biomechanics. For example, Noailly and Lacroix^1^ explored spine modeling methodologies, highlighting the need for improved validation techniques to ensure clinical applicability. Ahmadi et al.^2^ constructed a lumbar spine FEA model using detailed anatomical and material properties extracted from CT and MRI images, achieving over 94% accuracy in predicting key mechanical parameters such as Young’s modulus, Poisson’s ratio, bulk modulus, and shear modulus for both bone and disc tissues. In our work, we similarly developed an FEA model based on segmented and meshed vertebrae and discs from clinical imaging, with PINNs applied to ensure material predictions conformed to physical laws. Similarly, Paoli et al.^3^ investigated patient-specific FEA models, incorporating medical imaging data for enhanced accuracy. Additionally, explicit finite element modeling approaches have been employed to simulate high-impact conditions, as demonstrated by Firoozbakhsh et al.^4^. Further research has explored the role of intervertebral disc degeneration in spinal biomechanics, with Sun et al.^5^ reviewing the latest finite element models for intervertebral discs and their applications in understanding degenerative conditions. Zhang et al.^6^ applied FEA to study adolescent idiopathic scoliosis, providing insights into stress distribution and range of motion restrictions.

Lianget al.^7^ Song et al. developed an automated computational method using 3D point cloud registration and a Siamese PointNet++ network to enhance the precision of pedicle screw placement in spinal surgery, particularly for osteoporotic patients. Their approach improves accuracy and efficiency in screw trajectory planning from CT images, demonstrating strong potential for clinical application.Ahmadi et al.^8^ Ahmadi et al. review how Physics-Informed Machine Learning (PIML) integrates physical laws into data-driven models, enhancing interpretability and accuracy in medical imaging tasks such as reconstruction, segmentation, and anomaly detection. The review highlights recent advances, key challenges, and future directions for PIML across modalities such as MRI, CT, and ultrasound. Luan et al.^9^ proposed a unified density-modulus relationship for the human lumbar vertebral body, enhancing material property assignment in FEA models.

Recent studies have also explored the influence of muscle activity on spinal biomechanics. Ding et al.^10^ assessed the biomechanical effects of unilateral and bilateral lumbar spondylolysis using FEA, highlighting the role of muscle weakness in increasing range of motion and stress concentrations. Li et al.^11^ analyzed the quantitative relationships between elastic modulus and the biomechanical properties of transforaminal lumbar interbody fusion (TLIF), offering insights into optimal fixation strategies. Moreover, Zhu et al.^12^ developed and validated a finite element model to investigate how vibration affects the dynamic biomechanical response of the lumbosacral spine. Their findings highlight the significant impact of upper body mass and physiological spine curvature on vibration-induced stress, suggesting that local high stress in intervertebral discs is a key safety parameter for lumbar spine vibration assessment. Shastry et al.^13^ introduced deep learning-based spinal segmentation techniques that significantly reduce manual intervention, while Campbell et al.^14^ proposed automated meshing methods that enhance simulation accuracy. Caprara et al.^15^ demonstrated the integration of deep learning with FEA to improve patient-specific spinal modeling, paving the way for more efficient and clinically applicable simulations. Almalki et al.^16^ focused on vertebral endplate modeling using Principal Component Analysis (PCA), ensuring accurate geometric representation in computational modelsŁosiński et al.^17^ demonstrate that the non-invasive KINEOD device can detect significant changes in lumbosacral spinal alignment parameters following open discectomy. Sacral inclination angle (Sacral IA) proves to be a particularly reliable marker for rapid postoperative assessment of dynamic changes in the lumbar spine. Thibeault^18^ developed a framework using multiview Transformers and neural implicit fields to predict postoperative upright spine geometry in pediatric scoliosis patients undergoing AVT surgery. This model, trained in 652 cases and tested on 83, enables accurate 3D alignment forecasting from intraoperative imaging.

Xu et al.^19^ demonstrated through finite element analysis that OLIF combined with posterior bilateral percutaneous pedicle screw fixation (BPS/TINA) reduces interbody stress and spinal ligament tension more effectively than OLIF with a lateral plate system (LPS), thereby providing greater segmental stability. However, OLIF + BPS (TINA) may be more likely to increase the risk of adjacent segment degeneration compared to OLIF + LPS. Guo et al.^20^ developed an enhanced YOLOv8 model (GE-YOLOv8) for automated lumbar spine MRI interpretation, incorporating gradient search and efficient channel attention modules to boost feature extraction and detection accuracy. GE-YOLOv8 outperformed standard YOLOv8 with a 4.4% increase in mAP50 and a 2.1% reduction in parameters, achieving excellent results on internal and external datasets. El Bojairami et al.^21^ developed and validated a detailed finite element spine model that accurately represents major torso tissues, using a novel meshing approach to ensure computational efficiency. Validation against experimental data showed the model reliably simulates spinal mechanics and loadings, making it a valuable tool for biomechanical assessment.

Liang et al.^22^ proposed an automated process for lumbar spine FEA model development, aiming to enhance reproducibility and reduce manual intervention, aligning with the present study’s objectives of improving segmentation and meshing automation. The studies discussed the growing need for automated methodologies in spinal biomechanics. Ahmadi et al.^23^ introduced an FEA approach for lumbar spine biomechanics that integrates computational modeling to streamline the workflow from imaging to simulation. Their pipeline reduced model preparation time by 97.9%, cutting it from over 24 h to just 30 min and 49 s. Doulgeris et al.^24^ validated multiple lumbar spine finite element models using CT data from eight individuals, assessing the influence of age and gender on biomechanical outcomes. Their analysis confirmed that age is a key factor affecting model predictions, while gender had little impact on lumbar spine biomechanics.

**Fig. 1:**
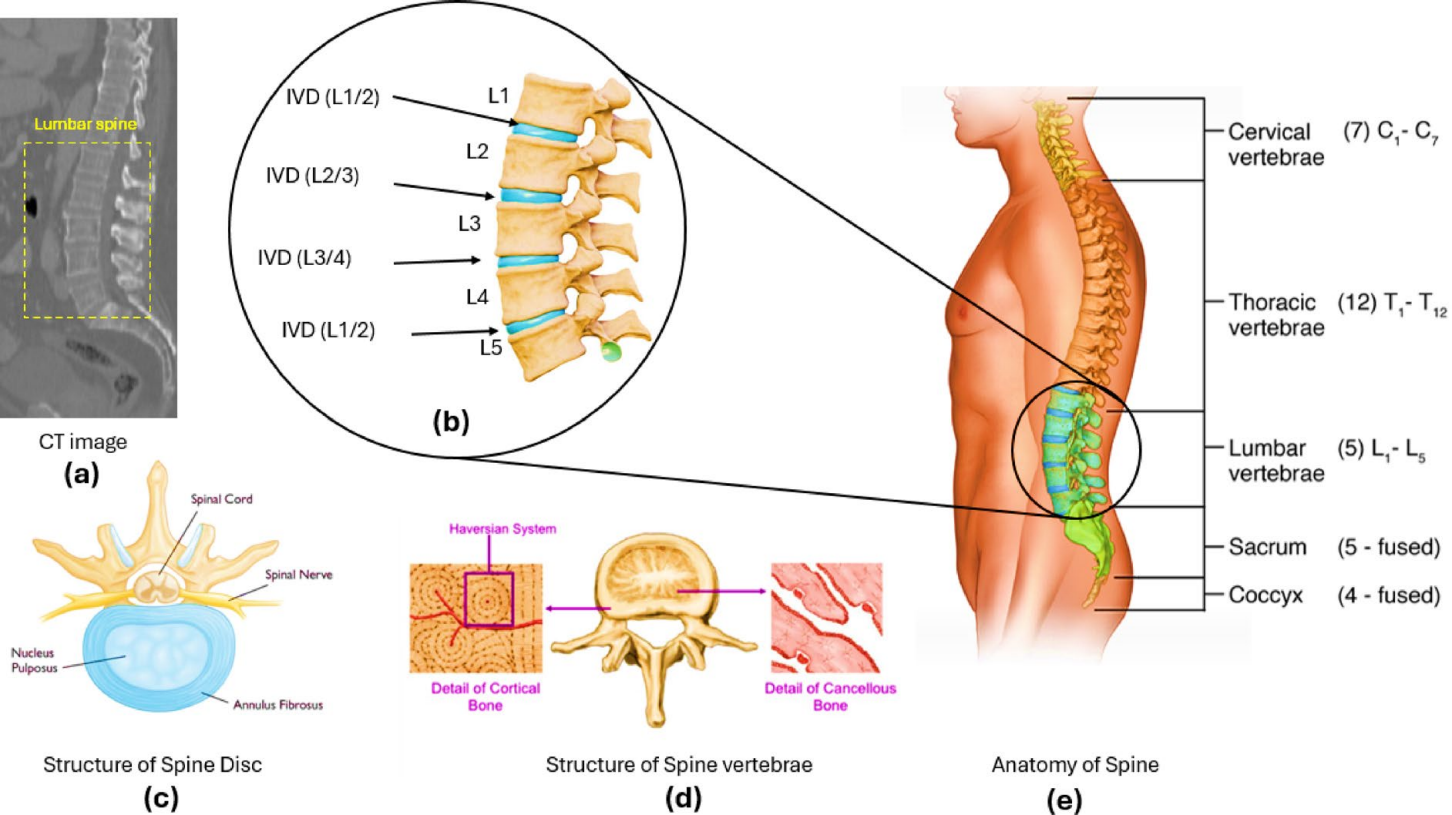
Anatomical and structural representation of the human spine. (**a**) CT scan of the lumbar spine, highlighting the lumbar region. (**b**) Zoomed-in view of the lumbar vertebrae (L1–L5) and intervertebral discs (IVD). (**c**) Structure of the intervertebral disc, showing the nucleus pulposus and annulus fibrosus. (**d**) Structure of the spine vertebrae, illustrating cortical and cancellous bone with the Haversian system. (**e**) Full anatomical categorization of the spine, distinguishing cervical, thoracic, lumbar, sacral, and coccygeal regions.

This Fig. 1 provides a comprehensive anatomical and structural overview of the human spine, with a focus on the lumbar region. (a) The left section displays a CT scan of the lumbar spine, highlighting the vertebral structures and intervertebral discs. (b) The central zoomed-in view illustrates the lumbar vertebrae (L1–L5) along with their corresponding intervertebral discs (IVD), which play a crucial role in load distribution and spinal flexibility. (c) The bottom-left diagram represents the structure of the intervertebral disc, showing the nucleus pulposus and annulus fibrosus. (d) The middle-bottom diagram highlights the structure of the vertebrae, detailing the cortical and cancellous bone, including the Haversian system. (e) The right section presents the full spinal column, categorizing it into cervical (C1–C7), thoracic (T1–T12), lumbar (L1–L5), sacrum (5 fused), and coccyx (4 fused) regions.

This study presents an automated methodology for generating anatomically accurate, subject-specific lumbar spine models optimized for FEA. The primary focus of our work is the development and automation of the preprocessing pipeline from medical image segmentation to the creation of simulation-ready meshes. Our approach integrates advanced computational tools, including the GIBBON library and FEBio, significantly reducing runtime and eliminating manual intervention throughout the model generation process. State-of-the-art deep learning frameworks are utilized for precise segmentation of vertebrae and intervertebral discs, which are subsequently converted into high-fidelity surface meshes and further optimized using Laplacian smoothing and geometric decimation. This methodology enables efficient extraction and meshing of cortical and cancellous bone, nucleus, annulus, cartilage, and ligaments, ensuring anatomically realistic models. Ligaments are modeled by automatically defining attachment points using spherical coordinate-based segmentation, while PCA is employed to extract vertebral endplates for accurate cartilage modeling. As a result, the automated pipeline achieves a substantial reduction in preparation time compared to traditional manual workflows, improving both efficiency and reproducibility.

Although the principal contribution of this study is the fully automated preprocessing workflow, we further demonstrate the compatibility and reliability of the generated models by conducting sample FEA simulations under physiological loading conditions. These sample simulations serve to validate that our automated models can effectively replicate the biomechanical behavior of the lumbar spine, matching or exceeding the performance of conventional approaches. Recent advancements in computational methodologies have enabled the development of subject-specific spinal finite element models with improved efficiency. For example, Hadagali et al.^25^ employed a semi-automated approach combining dual-kriging with multi-block meshing to create patient-specific models for scoliosis analysis. While this method successfully reduced some manual labor, it still required significant user interaction for boundary definition and mesh refinement, leading to workflow variability and time-consuming processes. Other methods have focused on automated segmentation of vertebral bodies but still rely on manual meshing of complex intervertebral discs and ligaments. Our study bridges this gap by presenting a fully automated pipeline for lumbar spine FEA modeling, including segmentation, meshing, and ligament attachment thereby offering a substantial improvement in efficiency, reproducibility, and scalability over existing semi-automated approaches.

This approach provides a robust framework for advancing spinal biomechanics research, with broad applications in clinical diagnostics, implant design, and rehabilitation planning. The automated pipeline introduced in this study enhances reproducibility and scalability, addressing limitations in current FEA methodologies and paving the way for real-time biomechanical simulations in both research and clinical contexts.

## Methods and materials

### Proposed automated method

In this study, we utilized FEA to investigate the biomechanical behavior of the lumbar spine. The modeling process began with CT image segmentation and progressed through the automatic extraction of cortical bone, cancellous bone, annulus, nucleus, cartilage, and ligaments, all implemented using advanced computational techniques. Specifically, we developed a custom pipeline in MATLAB, leveraging the GIBBON library and FEBio for precise simulations. This section details the methodology for the automated extraction and meshing of cortical and cancellous bone structures, which are integral to the analysis. The process of extracting the vertebrae and intervertebral discs from CT images involves advanced segmentation methods that utilize state-of-the-art deep learning algorithms. This section describes the workflow for generating Standard Tessellation Language (STL) files or surface representations of the vertebrae and discs, which are critical for biomechanical modeling and FEA. The dataset used in this study consisted of a CT scan from a subject obtained from the publicly available Large Scale Vertebrae Segmentation Challenge dataset^26^. Our automated pipeline was developed and implemented using MATLAB (R2024b), leveraging its built-in functions and the GIBBON toolbox for geometry processing and meshing. Finite element simulations were performed using FEBio (v2.5). For the deep learning-based segmentation, we employed the pre-trained TotalSegmentator (v2.7.0) package, which is built on the nnUNet and MONAI frameworks. The segmentation process was accelerated using GPU acceleration, which is a key factor in achieving the pipeline’s high efficiency. The hardware used for running the pipeline included an Intel Core i7 processor, 16 GB of RAM, and an NVIDIA GeForce RTX 3060 with 8 GB of VRAM, which provides a suitable configuration for both the deep learning inference and the subsequent computational steps.

The CT images used for the extraction process were first preprocessed to ensure optimal input for the segmentation models. This preprocessing included resampling the image volumes to isotropic spacing and normalizing the intensity values. Such preprocessing steps reduce variability across datasets and enhance the performance of the segmentation algorithms. The vertebrae and discs were analyzed separately due to their distinct anatomical features and segmentation requirements. For the segmentation of vertebrae and intervertebral discs, we leveraged the TotalSegmentator package, a state-of-the-art, pre-trained network based on the nnUNet framework. The use of this pre-trained model is a key aspect of our automated workflow, as it circumvents the need for a custom, large-scale training dataset and a time-consuming training phase. TotalSegmentator was originally trained on a diverse imaging dataset of over 1200 CT scans, encompassing a broad range of anatomical variations, patient ages, and pathologies. This robust training allows our pipeline to accurately and reliably segment lumbar spine structures, regardless of variations in patient anatomy or the presence of common pathologies such as osteophytes or mild degenerative changes. By integrating this pre-trained and validated network, our methodology ensures high-quality segmentation while maintaining the core principle of a fully automated process with minimal manual intervention. The nnUNet framework underlying TotalSegmentator is a self-configuring deep learning segmentation system that automatically adjusts its hyperparameters, network architecture, and preprocessing steps based on the input dataset.

**Fig. 2:**
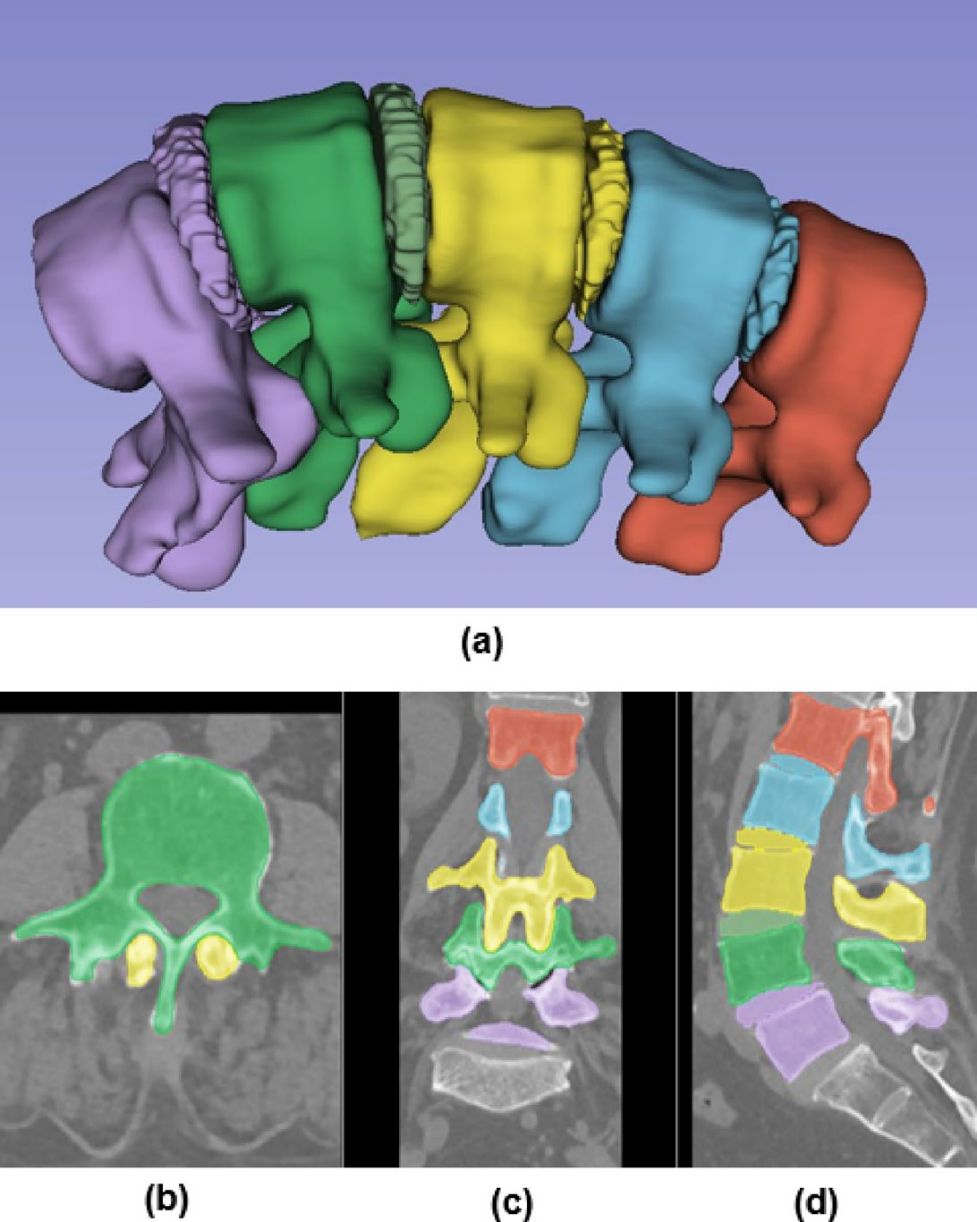
Automated segmentation and 3D visualization of the lumbar spine. (**a**) Reconstructed 3D model with color-coded vertebrae, intervertebral discs, and associated structures. (**b**) Axial view of the segmented CT image, showing vertebrae and intervertebral discs. (**c**) Coronal view with labeled vertebrae and intervertebral discs. (**d**) Sagittal view of the segmented spine, processed using nnUNet for segmentation and MONAI for labeling, color mapping, and refinement.

 The segmentation process involved identifying regions of interest (ROIs) around the spine and applying nnUNet’s 3D U-Net architecture to segment the discs at each level (L1/L2, L2/L3, etc.). Post-segmentation, a morphological smoothing step was applied to ensure the continuity and anatomical accuracy of the disc boundaries. The segmentation results were then processed using MONAI, which assigned distinct labels, applied color mapping, and refined the segmented structures for visualization. This approach allowed for precise extraction of vertebras and discs. The final labeled and color-coded segmentation results were converted into 3D meshes, facilitating further biomechanical analysis and FEA simulations (see Fig. 2). The segmented regions were converted into 3D surface meshes (STL files) using 3D slicer. These STL files represent the discs with high fidelity and are ready for further computational modeling.

For the vertebrae, the segmentation was performed using the MONAI framework, a specialized library for medical imaging applications. MONAI leverages deep learning models, such as 3D U-Net, combined with data augmentation techniques to segment complex anatomical structures like the vertebrae. The segmentation pipeline began with ROI localization to isolate the vertebral column from surrounding tissues. A pre-trained 3D U-Net model was then fine-tuned on the CT dataset to segment each vertebra. The segmentation results were post-processed to remove noise and small artifacts. Specifically, connected component analysis was applied to retain only the largest connected regions, corresponding to the vertebrae. Following segmentation, the surface geometry of the vertebrae was reconstructed. The segmented volumetric masks were converted into STL files using marching cubes algorithms, which extract isosurfaces from volumetric data. To ensure smooth and anatomically accurate surfaces, Laplacian smoothing and decimation techniques were employed. These steps reduce mesh complexity without compromising anatomical detail, making the STL files suitable for biomechanical analysis.

### Cortical and cancellous extraction

The vertebral body is a composite structure, and for accurate biomechanical modeling, it must be differentiated into its primary components: the dense, outer cortical bone and the porous, inner cancellous bone. The cortical bone is modeled as the outer shell of the vertebrae, providing the primary structural resistance to mechanical loads, while the cancellous bone represents the internal, spongy trabecular structure. In this work, the cortical layer thickness is assumed to be uniform at $t_{{\text{core }}} = 0.3{\text{ mm}}$, and the smoothing factor $\left( \lambda \right)$ for the core vertices is constrained to the interval $0 < \lambda \le 1$. The extraction process involves several computational steps designed to ensure geometric accuracy and structural integrity, which we will now formally describe.

Initially, the geometry of each vertebra (L1 to L5) is loaded. This geometry is represented as a triangular mesh, ${\mathcal{M}}$, which is formally a tuple ${\mathcal{M}} = \left( {V,F} \right)$, where $V$ is a set of vertex coordinates in ${\mathbb{R}}^{3}$, and $F$ is a set of triangular faces defined by triplets of indices into $V$. The first step in differentiating the cancellous core from the cortical shell is to determine the direction of the inward offset. This is achieved by computing the normal vector for each triangular face. For a given face $f \in F$ defined by the ordered vertices $\left( {v_{1} ,v_{2} ,v_{3} } \right)$, where $v_{i} \in V$, two edge vectors, ${\mathbf{e}}_{1}$ and ${\mathbf{e}}_{2}$, coplanar with the face are defined as ${\mathbf{e}}_{1} = v_{2} - v_{1}$ and ${\mathbf{e}}_{2} = v_{3} - v_{1}$. The vector cross product of these edges yields a vector perpendicular to the face plane. To ensure this vector represents a unit direction, it is normalized by its Euclidean norm. The normals for each triangular face are computed using the formula:

1 $${\mathbf{N}}_{f} = \frac{{\left( {{\mathbf{v}}_{2} - {\mathbf{v}}_{1} } \right) \times \left( {{\mathbf{v}}_{3} - {\mathbf{v}}_{1} } \right)}}{{\left. {\parallel {\mathbf{v}}_{2} - {\mathbf{v}}_{1} } \right) \times \left( {{\mathbf{v}}_{3} - {\mathbf{v}}_{1} } \right)\parallel }}$$

where ${\mathbf{v}}_{1} ,{\mathbf{v}}_{2} ,{\mathbf{v}}_{3}$ are the vertices of a given face. These normals guide the inward displacement of vertices during the creation of the cancellous core. To generate the core, the vertices are moved inward by the specified core thickness along the normal vectors.$V_{{\text{cortical }}}$, is translated along the direction opposite to its corresponding face normal, $N_{f}$. To generate the core, the vertices are moved inward by the specified core thickness along the normal vectors. Mathematically, the new vertex positions $\left. {(V_{{\text{cancellous }}} } \right)$ are obtained through a vector translation operation given by:

2 $${\mathbf{V}}_{{\text{cancellous }}} = {\mathbf{V}}_{{{\text{cortical}}}} - t_{{\text{core }}} \cdot {\mathbf{N}}_{f}$$

**Fig. 3:**
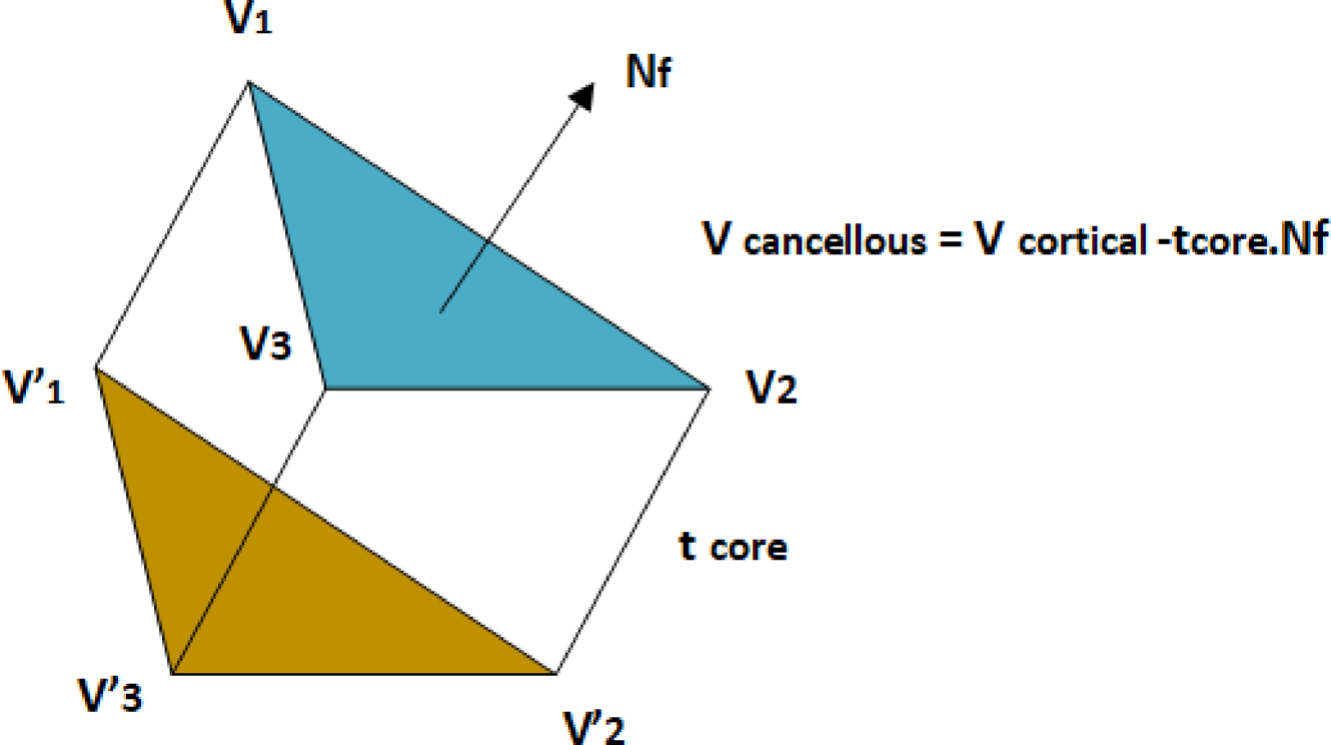
Illustration of the inward displacement of cortical vertices $\left( {V_{1} ,V_{2} ,V_{3} } \right)$ to generate cancellous vertices $\left( {V_{1}{\prime} ,V_{2}{\prime} ,V_{3}{\prime} } \right)$ based on the face normal $\left( {N_{f} } \right)$ and core thickness $\left( {t_{{\text{core }}} } \right)$. The transformation follows the formula $V_{{\text{cancellous }}} = V_{{\text{cortical }}} - t_{{\text{core }}} \cdot N_{f}$.

$t_{{\text{core }}}$ represents the core thickness. This linear translation can introduce sharp features and selfintersections into the newly generated cancellous mesh. Following this, a Laplacian smoothing algorithm is applied iteratively to the core vertices to eliminate these artifacts and regularize the geometry. Laplacian smoothing repositions each vertex based on the average position of its neighbors, effectively acting as a low-pass filter on the mesh geometry. Figure 3 illustrates the inward displacement of cortical vertices to generate cancellous bone geometry using the face normal direction and core thickness. The transformation formula ensures accurate differentiation between cortical and cancellous regions in the automated meshing process. During each iteration of the smoothing process, the updated position for a vertex $v_{i}$ is computed as a weighted average of its current position and the centroid of its neighboring vertices:

3 $${\mathbf{v}}_{i}^{{\text{new }}} = \left( {1 - \lambda } \right) \cdot {\mathbf{v}}_{i} + \lambda \cdot \frac{{\sum\limits_{{j \in {\mathcal{N}}\left( i \right)}} {{\mathbf{v}}_{j} } }}{{\left| {{\mathcal{N}}\left( i \right)} \right|}}$$

$\mathcal{N}(i)$ denotes the set of neighboring vertices of $i$ (i.e., the 1-ring neighborhood), $|\mathcal{N}(i)|$ is the cardinality of this set (the vertex valence), and $\lambda$ is a relaxation parameter that controls the smoothing intensity. The cortical and cancellous meshes are subsequently remeshed using the Vorpalite tool, part of the Geogram library, which performs variational anisotropic surface meshing to ensure high-quality meshes suitable for FEA. This remeshing process aims to achieve a specific vertex density or edge length while preserving geometric features. For volumetric meshing, the Partial Differential Equation (PDE) Toolbox in MATLAB is utilized. The meshes are refined to create a hollow shell for the cortical bone by removing internal elements corresponding to the cancellous part. This Boolean operation ensures the integrity of the cortical shell as a thin layer surrounding the cancellous core. The resulting meshes are saved in multiple formats, including MATLAB (.mat) and VTK, for further processing and visualization. The automated extraction and meshing methodology ensures consistency across vertebrae (L1 to $\text{L}5)$, providing a robust basis for subsequent FEA simulations. These simulations involve the application of various mechanical loads on the lumbar spine, enabling detailed analysis of its biomechanical properties.

**Algorithm 1:**
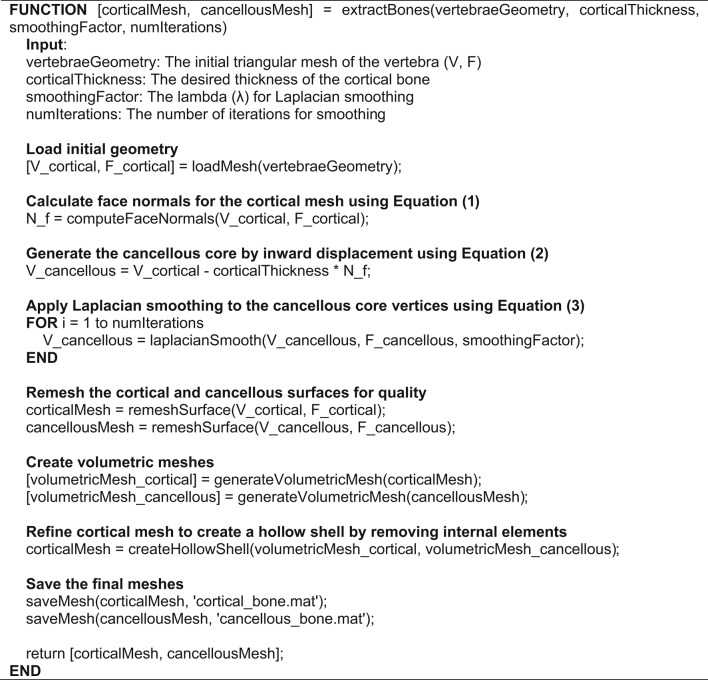
Main function for cortical and cancellous extraction

**Fig. 4:**
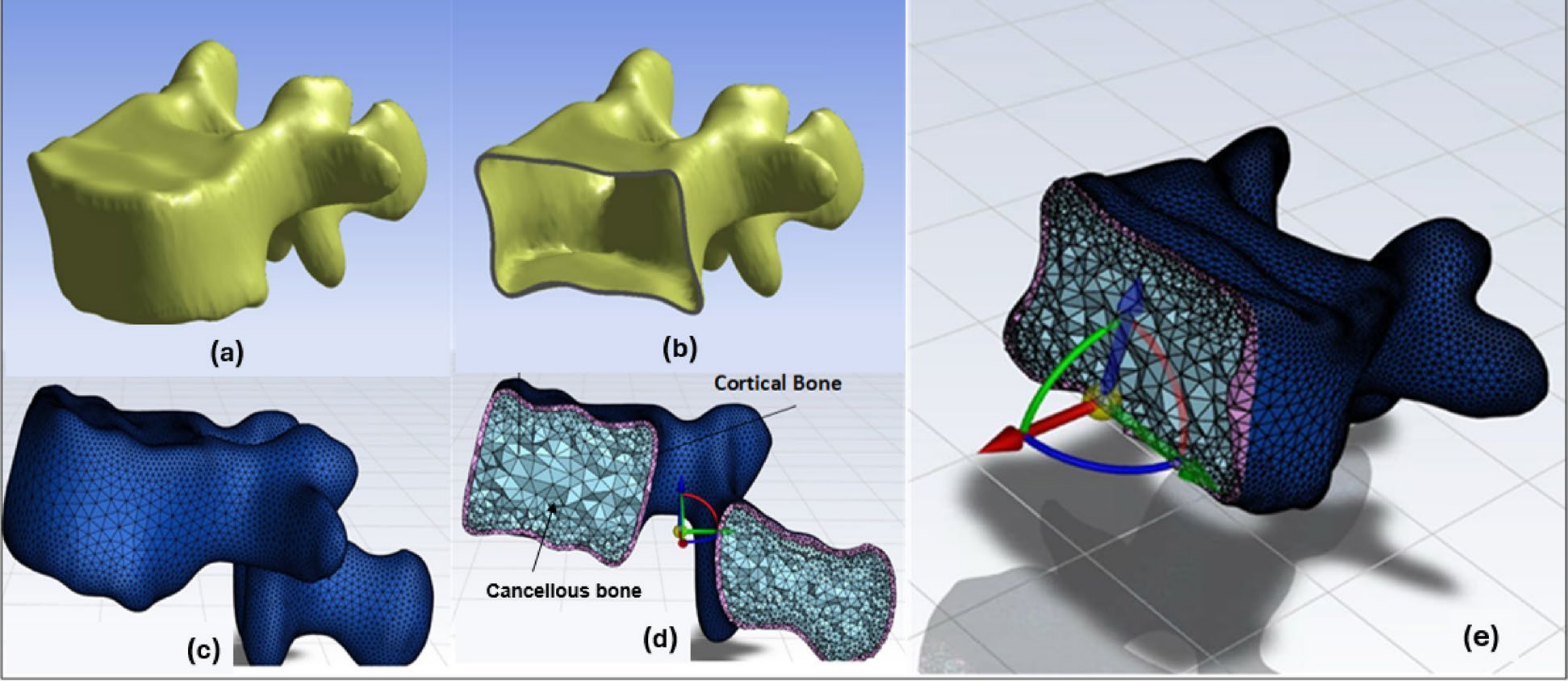
Mesh generation of a lumbar vertebra, illustrating the segmentation and meshing process. (**a**) Extracted vertebral geometry from CT segmentation. (**b**) Processed vertebra with a defined inner boundary for cortical and cancellous separation. (**c**) Surface mesh representation of the vertebra. (**d**) Cross-sectional view highlighting the distinction between cancellous and cortical bone. € Integrated finite element mesh with applied boundary conditions, demonstrating the preparation for FEA simulations.

Manual meshing of the vertebrae and discs is prohibitively time-consuming and prone to user-dependent errors. Our automated pipeline addresses this challenge by leveraging the robust capabilities of automatic meshing tools, specifically implemented within the GIBBON library and MATLAB’s PDE toolbox. These tools are designed to generate high-fidelity tetrahedral meshes from complex geometries. While the manuscript does not explicitly report mesh quality metrics (such as aspect ratio, skewness, or warping), the automated meshing process incorporates internal algorithms and parameters that optimize these metrics to ensure the mesh is of sufficient quality for accurate biomechanical simulations. This approach ensures consistency and reproducibility, representing a significant improvement over manual methods, where mesh quality can vary considerably. Figure 4 presents the automated segmentation and meshing workflow for a lumbar vertebra, highlighting the distinction between cortical and cancellous bone structures.

The use of automated geometry extraction, smoothing, and remeshing techniques minimizes manual intervention, enhancing the reproducibility and accuracy of the study.

### Defining nucleus geometry using PCA

In this study, the robust separation and high-fidelity meshing of the annulus fibrosus and nucleus pulposus geometries are fundamental to developing a biomechanically accurate model of the lumbar spine’s intervertebral disc. The process begins by algorithmically defining the nucleus pulposus geometry related to the enclosing annulus fibrosus, utilizing geometric analysis and logical set operations. After this initial separation, both geometries undergo refinement steps to achieve precise alignment, surface smoothness, and topological completeness removing any artifacts that may arise during automated generation. These steps are critical to preparing the models for advanced FEA and employ sophisticated computational methods for the separation, meshing, and precise alignment of the nucleus and annulus. To initialize the nucleus geometry, the vertices of the intervertebral disc (annulus) are first projected onto a plane derived from PCA.

PCA is a statistical procedure that uses an orthogonal transformation to convert a set of observations of possibly correlated variables into a set of values of linearly uncorrelated variables called principal components. In the context of disc geometry, PCA is an effective method for identifying the intrinsic orientation of the disc and defining an optimal plane for vertex projection. This is achieved by identifying the directions of maximum and minimum variance of the vertex coordinates. Let the set of all vertices of the disc geometry be ${\mathbf{V}} = \left\{ {v_{1} ,v_{2} , \ldots ,v_{n} } \right\}$, where $v_{i} = \left( {x_{i} ,y_{i} ,z_{i} } \right)$ are the coordinates. The first step is to center the data by subtracting the mean vertex position ${\overline{\mathbf{v}}}$ from each vertex:

4 $${\mathbf{v}}_{i}{\prime} = {\mathbf{v}}_{i} - {\overline{\mathbf{v}}}{ }\;{\text{where }}\;{\overline{\mathbf{v}}} = \frac{1}{n}\sum\limits_{i = 1}^{n} {{\mathbf{v}}_{i} }$$

Next, the covariance matrix ${{\varvec{\Sigma}}}$ is computed from the centered data. This matrix encapsulates the geometric variance and relationships between the coordinate axes.

5 $${{\varvec{\Sigma}}} = \frac{1}{n - 1}\sum\limits_{i = 1}^{n} {\left( {{\mathbf{v}}_{i}{\prime} } \right)} \left( {{\mathbf{v}}_{i}{\prime} } \right)^{T}$$

The principal components are the eigenvectors (${\mathbf{u}}_{1} ,{\mathbf{u}}_{2} ,{\mathbf{u}}_{3}$) of the covariance matrix, and the corresponding eigenvalues ($\lambda_{1} ,\lambda_{2} ,\lambda_{3}$) represent the variance along each component. The eigenvectors and eigenvalues are found by solving the characteristic equation:

6 $${\mathbf{\Sigma u}}_{k} = \lambda_{k} {\mathbf{u}}_{k}$$

The first principal component $({\mathbf{u}}_{1} )$ corresponds to the direction of maximum variance (the longest axis of the disc), and the third $({\mathbf{u}}_{3} )$ corresponds to the direction of minimum variance (the disc’s central axis, which is the direction of its smallest dimension). The projection plane for the nucleus boundary is then defined by the first two principal components, ${\mathbf{u}}_{1}$ and ${\mathbf{u}}_{2}$, which are orthogonal to the disc’s central axis $({\mathbf{u}}_{3} )$.

The projected boundary of the disc is then shrunk using a user-defined scaling factor $(S)$ to define the nucleus boundary. This rescaled curve, $C_{{\text{nucleus }}}$, is a scaled version of the disc boundary, $C_{{\text{disc }}}$.

7 $$C_{Nucleus} = s \cdot C_{{\text{Disc }}}$$

The nucleus geometry was remeshed using a specified point spacing to ensure uniformity and consistency across the surface.

### Nucleus and annulus extraction

The function *meshInsideGeometry* generated internal elements for the nucleus geometry, allowing the model to prepare for interaction with the annulus. To align the nucleus with the annulus, centroids of both geometries were computed as:

8 $${\text{ Centroid }}_{{\text{Nucleus }}} = \frac{1}{n}\sum\limits_{i = 1}^{n} {V_{{{\text{Nucleus }}1,i}} }$$

9 $${\text{Centroid }}_{{\text{Annulus }}} = \frac{1}{m}\sum\limits_{j = 1}^{m} {V_{{{\text{Annulus }}1,j}} }$$

where $n$ and $m$ are the number of vertices in the nucleus and annulus, respectively. The nucleus geometry was translated to align its centroid with that of the annulus. Using the function inpolyhedron, vertices of the disc geometry were checked for their inclusion in the nucleus geometry, and a logical array flagged whether each vertex was inside or outside the nucleus. The disc geometry was then separated into annulus and nucleus regions. Faces of the disc geometry $(F_{{\text{Disc }}} )$ with all vertices outside the nucleus were classified as annulus faces, while those with at least one vertex inside the nucleus were classified as nucleus faces. Mathematically, for a face $f$:

- Annulus: $$f \in \left\{ {F_{Disc } \left| {\forall v_{i} \notin V_{Nucleus 1} } \right.} \right\}$$
- Nucleus: $$f \in \left\{ {F_{Disc } \left| {\exists v_{i} \in V_{Nucleus1 } } \right.} \right\}$$

To further refine the nucleus, the top and bottom faces were identified using the Z-coordinate of their centroids.

**Algorithm 2:**
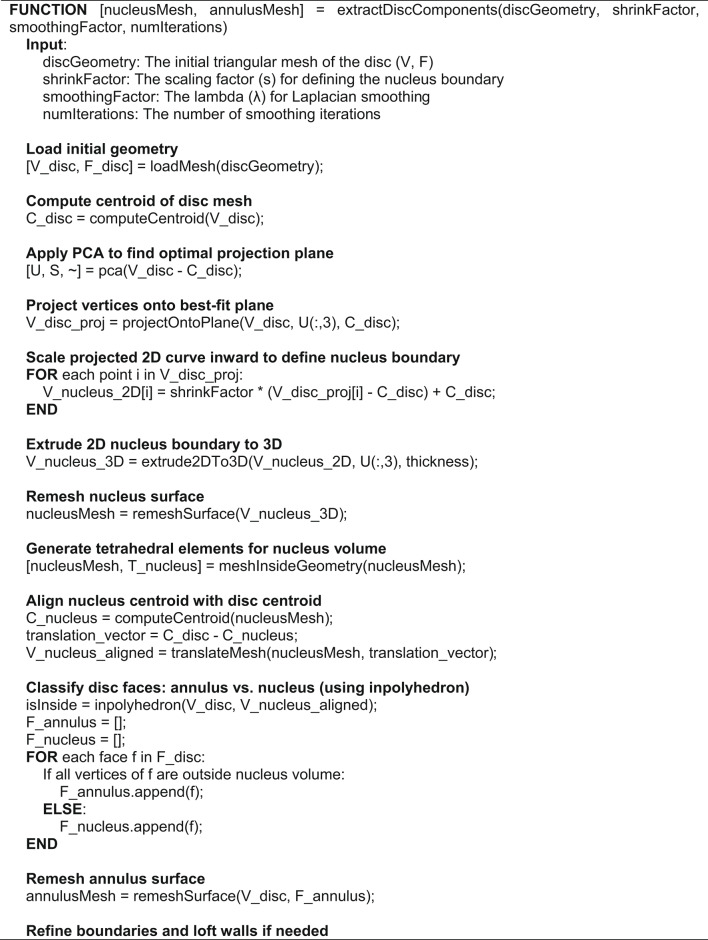
Main function for nucleus and annulus extraction

**Fig. 5:**
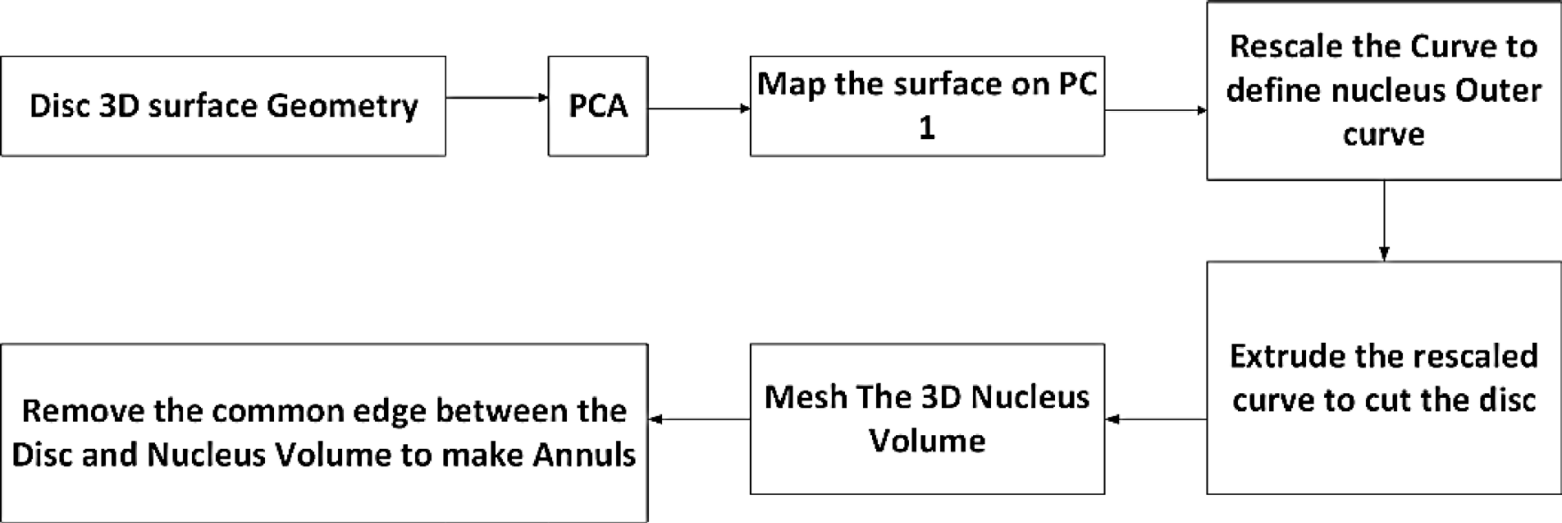
Workflow for separating and meshing the nucleus and annulus from the intervertebral disc geometry.

Figure 5 presents the process begins with the 3D surface mesh of the disc geometry, where PCA is applied to identify the principal components and map the surface onto the first principal plane. The nucleus is defined by rescaling the mapped curve using a scaling factor $(C_{{\text{Nucleus }}} = S \cdot C_{{\text{Disc }}} )$ and extruding the rescaled curve to create a 3D cylindrical nucleus. The nucleus volume is meshed, and the common edge between the disc and nucleus is removed to define the annulus geometry. The annulus is then meshed into a volumetric structure surrounding the nucleus.

A Z-threshold, calculated as the mean of the minimum and maximum Z-coordinates, was used to classify faces into top and bottom groups. The boundaries of these surfaces were extracted and resampled to ensure consistent point distribution. The lofting process then connected the top and bottom boundaries, forming the nucleus walls. This lofting was achieved by interpolating vertices between the top and bottom surfaces, generating a smooth quadrilateral mesh that was converted into triangles for consistency. The annulus geometry was processed similarly, ensuring that its boundaries aligned with the nucleus boundaries. The nearest neighbor mapping identified the corresponding annulus boundary points for each nucleus boundary point.

**Fig. 6:**
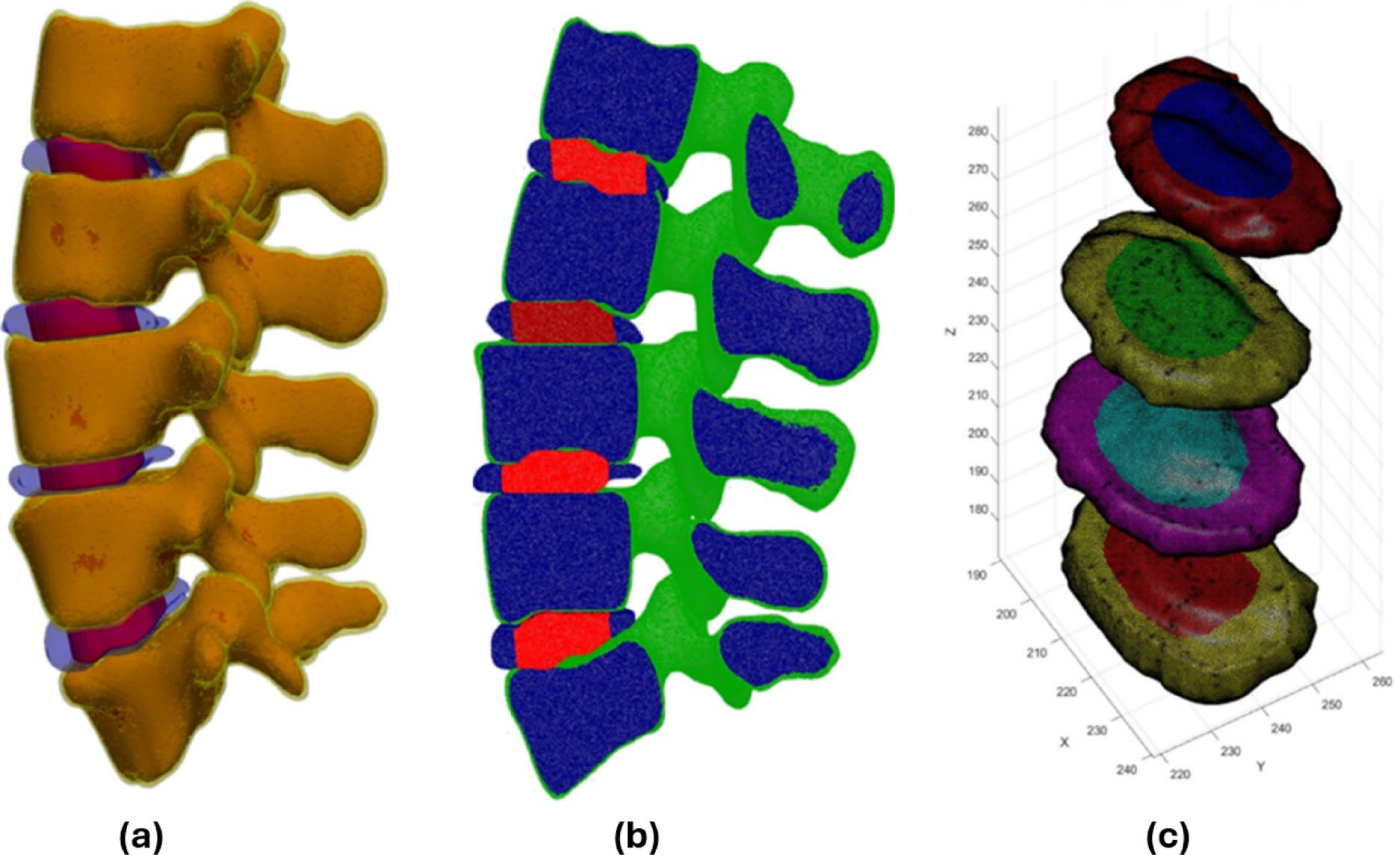
Automated segmentation and 3D visualization of the lumbar spine (L1–L5):(**a**) Detailed visualization of the segmented lumbar spine, with the annulus, nucleus, and cancellous regions colored separately.(**b**) Alternative view highlighting the spatial distribution of the segmented components. (**c**) 3D visualization of the nucleus and annulus geometries.These visualizations support accurate biomechanical modeling and FEA.

The annulus and nucleus walls were integrated by connecting their vertices and faces, creating a continuous structure. Finally, small holes in the meshes were filled using perimeter-based detection, and the internal regions of both geometries were meshed with tetrahedral elements using meshInsideGeometry. The outputs were cleaned and optimized to ensure compatibility with FEBio simulations, resulting in high-quality meshes of the annulus and nucleus. This methodical separation and meshing approach allowed for accurate biomechanical modeling and analysis of the lumbar spine. Figure 6 presents the automated segmentation of the lumbar spine (L1–L5), distinguishing the cortical, cancellous, nucleus, and annulus regions. The different views illustrate the three-dimensional structure of the segmented components, aiding in biomechanical modeling and FEA.

### Ligament modelling uses spherical coordination

In this study, ligament modeling was performed to connect the lumbar vertebrae (L1 to L5) using geometrically and anatomically accurate representations of various ligament types. The connections between the vertebrae were based on defined anatomical points derived from cylindrical coordinates, which were converted into Cartesian coordinates for accurate spatial placement. This modeling enabled the representation of key ligaments such as the anterior longitudinal ligament (ALL), intertransverse ligament (ITL), capsular ligament (CL), supraspinal ligament (SSL), posterior longitudinal ligament (PLL), and ligamentum flavum (LF). Each ligament was modeled by connecting corresponding anatomical regions on adjacent vertebrae.

**Fig. 7:**
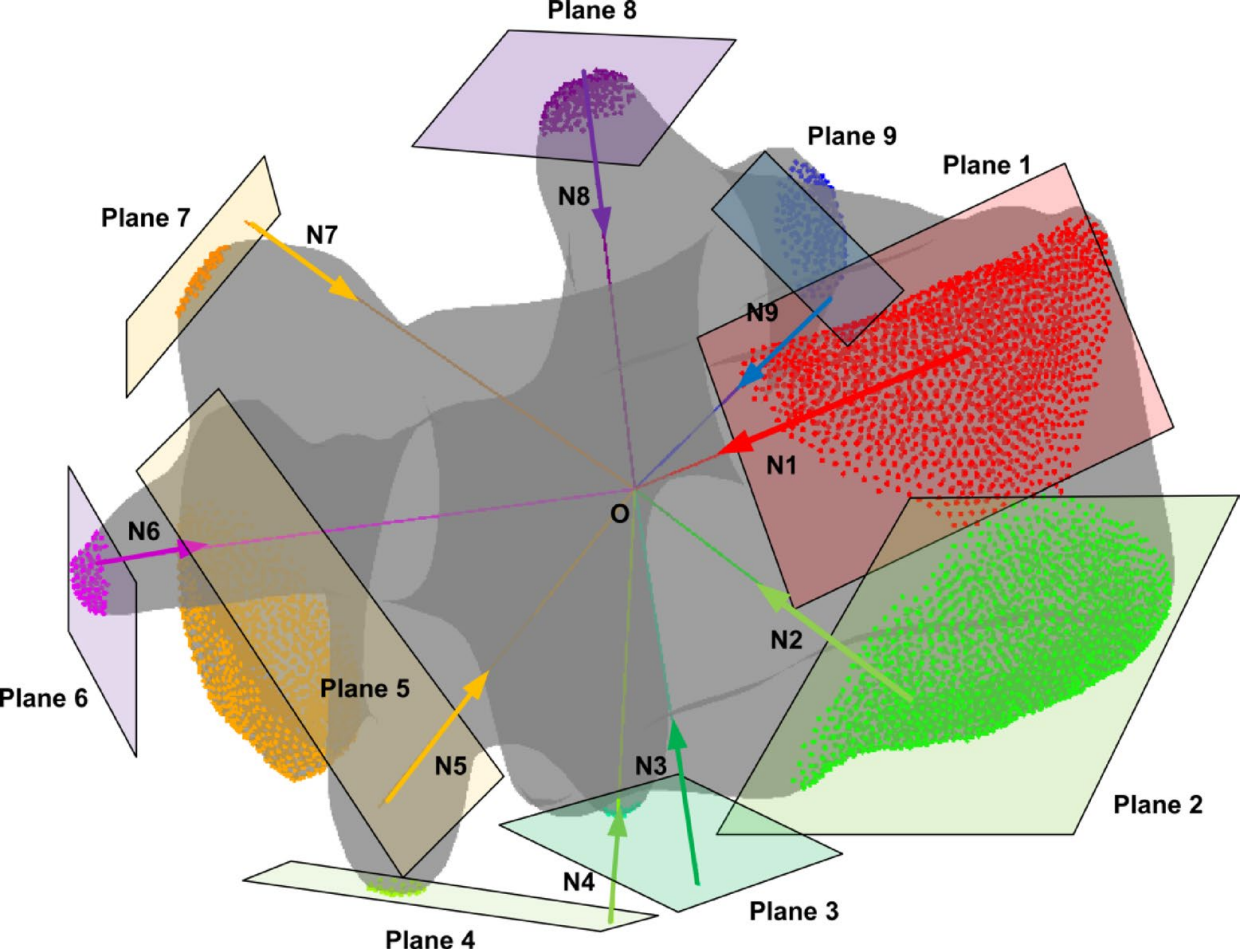
Automatic identification of ligament attachment areas on a vertebra using plane-based segmentation. The central point $O$ serves as the origin for spherical coordinates, with normal vectors ${N}_{1}$ to ${N}_{9}$ defining the orientation of planes (Plane 1 to Plane 9). Each plane divides the vertebral geometry into regions, with points on one side of the plane selected as ligament attachment areas.

Figure 7 illustrates the methodology used to specify areas on the vertebra for ligament connections by defining normal planes and identifying points of intersection with the vertebral geometry. This approach utilizes spherical coordinates to define the planes, and the vertices on the vertebra that lie on one side of each plane are automatically selected as ligament attachment points. The method ensures accurate spatial alignment of ligament attachment areas based on the vertebral anatomy. In this representation, the vertebra is analyzed by defining planes that pass through a central point $O$, which serves as the reference for the spherical coordinate system. Each plane is characterized by its normal vector, which is defined using spherical coordinates $(r,\theta ,\phi )$. The conversion from spherical coordinates to Cartesian coordinates for the normal vector $\text{N}$ is given by:

10 $${\mathbf{N}} = \left[ {\begin{array}{*{20}l} x \hfill \\ y \hfill \\ z \hfill \\ \end{array} } \right] = r\left[ {\begin{array}{*{20}c} {{\text{sin}}\left( \phi \right){\text{cos}}\left( \theta \right)} \\ {{\text{sin}}\left( \phi \right){\text{sin}}\left( \theta \right)} \\ {{\text{cos}}\left( \phi \right)} \\ \end{array} } \right]$$

The planes are then mathematically described by the equation:

11 $${\mathbf{N}} \cdot \left( {{\mathbf{P}} - {\mathbf{O}}} \right) = 0$$

where $\text{P}$ represents a point on the plane, $\text{O}$ is the origin point (center of the vertebra), and $\text{N}$ is the normal vector. This equation ensures that the plane is oriented in space based on the specified spherical coordinates. Each plane divides the 3D space into two regions: the positive and negative sides relative to the normal vector direction. To identify ligament attachment points, the vertices of the vertebral geometry are tested against the plane equation. If a vertex satisfies the inequality:

12 $${\mathbf{N}} \cdot \left( {{\mathbf{P}}_{{\text{vertex }}} - {\mathbf{O}}} \right) > 0$$

Then the vertex is considered to lie on the specified side of the plane and is included in the attachment area. This process is repeated for each plane, corresponding to specific ligament types. This method is fully automated, as the plane definitions and point selections are performed programmatically. The vertices on the vertebral geometry are tested for their position relative to each plane, and the corresponding ligament attachment areas are highlighted. The approach ensures that the identified regions accurately represent the spatial anatomy of the vertebra, providing a reliable foundation for subsequent ligament modeling. The anterior longitudinal ligament (ALL) was modeled by connecting the lower anterior point ${\text{(ALL}}_{{\text{up }}} )$ of the upper vertebra to the upper anterior point ${\text{(ALL}}_{{\text{down }}} )$ of the adjacent lower vertebra. This linear connection was established using the coordinates:

13 $${\mathbf{P}}_{{{\text{ALL}}}} = \left[ {\begin{array}{*{20}l} {x_{{\text{upper }}} } \hfill & {y_{{\text{upper }}} } \hfill & {z_{{\text{upper }}} } \hfill \\ {x_{{\text{lower }}} } \hfill & {y_{{\text{lower }}} } \hfill & {z_{{\text{lower }}} } \hfill \\ \end{array} } \right]$$

Similarly, the intertransverse ligament (ITL) was represented on the left and right transverse processes. For each side, ${\text{ITL}}_{{\text{left }}}$ and ${\text{ITL}}_{{\text{right }}}$ were connected between the upper and lower vertebrae, maintaining symmetry. Capsular ligaments $\left( {{\text{CL}}} \right)$ were modeled by connecting the lower capsular attachment points $\left( {{\text{CL}}_{{\text{low }}} } \right)$ on the lower vertebra to the upper capsular attachment points ${\text{(CL}}_{{{\text{up}}}} )$ on the upper vertebra. These connections were defined for both left and right attachment points. The coordinates for each point were mapped as follows:

14 $${\mathbf{P}}_{{{\text{CL}}}} = \left[ {\begin{array}{*{20}c} {x_{{\text{low }}} } & {y_{{\text{low }}} } & {z_{{\text{low }}} } \\ {x_{{\text{up }}} } & {y_{{\text{up }}} } & {z_{{\text{up }}} } \\ \end{array} } \right]$$

For the posterior longitudinal ligament (PLL), the geometry of the vertebral column was analyzed to identify the closest N points around the vertebral center, which were then connected to form the ligament structure. The ligamentum flavum (LF) was modeled by connecting midpoints between adjacent vertebral centers and the region around the supraspinal ligament (SSL).

The parameters in Table 1 represent the cylindrical coordinates for the attachment points of each ligament. These coordinates define the spatial locations of the ligaments in relation to the vertebrae, ensuring precise modeling of their biomechanical roles. The anterior longitudinal ligament (ALL) is represented by two points: the lower anterior point ${\text{(ALL}}_{{\text{down }}} )$ of the upper vertebra and the upper anterior point $\left( {{\text{ALL}}_{{{\text{up}}}} } \right)$ of the adjacent lower vertebra. These coordinates facilitate accurate connections along the anterior surfaces of the vertebrae, capturing the ligament’s role in anterior stabilization. The intertransverse ligament (ITL) is modeled by connecting the lateral regions of the transverse processes. Specifically, the left transverse process is defined by ${\text{ITL}}_{{\text{left }}}$, while the right is defined by ${\text{ITL}}_{{\text{right }}}$. These points ensure the proper representation of the ITL’s function in stabilizing lateral movement. The capsular ligaments $\left( {{\text{CL}}} \right)$ are represented by four points: ${\text{CL}}_{{{\text{lown\_left }}}} ,{\text{CL}}_{{{\text{lown\_right, }}}} ,{\text{CL}}_{{{\text{up\_left }}}}$, and ${\text{CL}}_{{{\text{up\_right}}{. }}}$. These points correspond to the attachment regions on the articular processes of adjacent vertebrae. By defining both the upper and lower attachment points on the left and right sides, the CL is accurately modeled as it connects vertebral joints and contributes to overall stability. The supraspinal ligament (SSL) is defined by $\text{SSL}$, which represents the ligament’s attachment along the spinous processes. This ligament provides posterior stabilization, particularly during flexion and extension of the spine. The posterior longitudinal ligament (PLL) and ligamentum flavum (LF) are modeled using central posterior and elastic connections, respectively.

**Table 1:** Parameters defining ligament attachment points on the lumbar spine, including their cylindrical coordinates, and anatomical descriptions.

Ligament	Point name	Cylindrical coordinates ($\phi ,\theta ,r$)
Anterior longitudinal (ALL)	Anterior longitudinal lower	(29.063, − 85, 18)
	Anterior longitudinal upper	(30, − 85, − 8)
Intertransverse (ITL)	Intertransverse left	(35, 30, − 1)
	Intertransverse right	(35, 156, − 1)
Capsular (CL)	Capsular lower left	(25, 128.63, 16)
	Capsular lower right	(18, 61, 21)
	Capsular upper left	(− 4, 30, − 28)
	Capsular upper right	(− 4, 140, − 27)
Supraspinal (SSL)	Supraspinal and interspinous ligament	(25, 90.7, − 30)
Posterior longitudinal (PLL)	Posterior longitudinal ligament	Closest N points to vertebral center
Ligamentum flavum (LF)	Ligamentum flavum	Midpoints between vertebrae centers

**Fig. 8:**
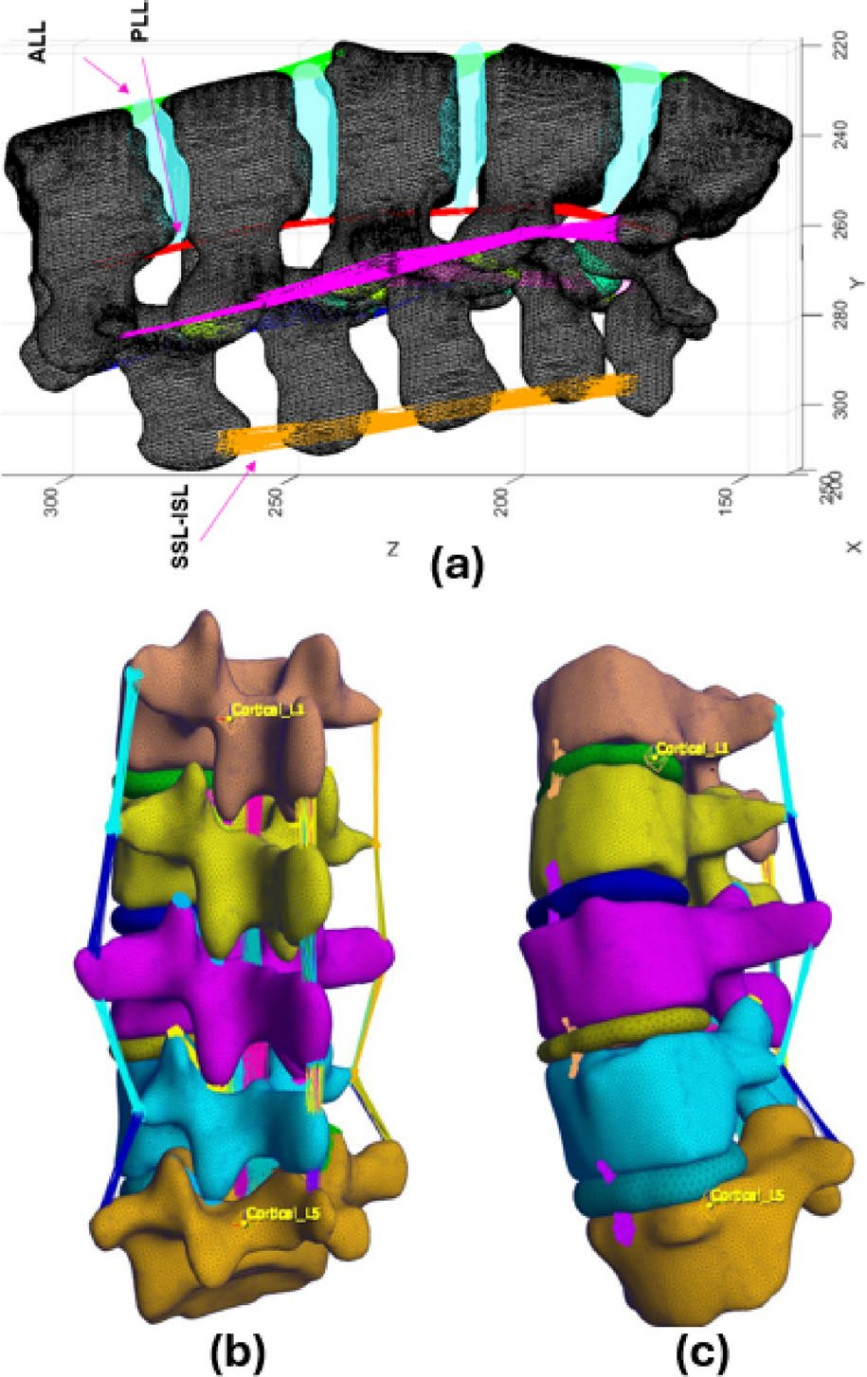
Results of ligament connections in the lumbar spine model, showing the spatial arrangement of key ligaments: ALL, PLL, SSL, ISL, CL, ITL, and LF. The ligaments are automatically modeled and visualized between adjacent vertebrae.

For the PLL, the N closest points around the vertebral center are identified and connected to create a continuous posterior ligament. The LF is modeled by linking the midpoints between adjacent vertebral centers to ensure elastic support and posterior arch stability. Figure 8 illustrates the automated ligament modeling in the lumbar spine, showing key ligament connections (ALL, PLL, SSL, ISL, CL, ITL, and LF) between adjacent vertebrae. The spatial arrangement of the ligaments is visualized to highlight their biomechanical role in spinal stability.

### Extraction of upper and lower endplate of the vertebrae

In this study, the upper and lower endplates of the lumbar vertebrae ($1$ to $L5$) were extracted and visualized using a detailed computational approach. The process involved the analysis of 3D mesh geometry, represented by vertices $(V)$ and faces $(F)$, to identify the regions corresponding to the vertebral endplates. This automated method uses PCA, and geometric shrinkage techniques to accurately isolate and refine the endplate regions. The vertebral centroid was first computed as the average position of all vertices, given by:

15 $${\mathbf{C}} = \frac{1}{n}\sum\limits_{i = 1}^{n} {{\mathbf{V}}_{i} }$$

where ${\text{V}}_{i}$ represents the 3D coordinates of the $i$-th vertex, and $n$ is the total number of vertices. The centroid served as the reference for centering the vertices prior to PCA. PCA was then applied to determine the principal axes of the vertebra. The direction of least variance, identified as the third principal component, was used to define the “light direction” $(\text{L})$, which distinguishes the top and bottom regions of the vertebra. To compute the face normal, the cross product of two edges of each triangular face was calculated using Eq. (1). where ${\text{V}}_{1},{\text{V}}_{2},{\text{V}}_{3}$ are the vertices of the triangle. The dot product of these normals with the light direction determined whether the faces were upward facing (positive dot product) or downward facing (negative dot product). The extraction of the upper endplate involved identifying vertices that belonged to upward-facing faces and were located above a threshold in the $z$-direction. This threshold was defined as:

16 $$z_{{\text{threshold }}} = \frac{{{\text{max}}\left( z \right) + {\text{min}}\left( z \right)}}{2}$$

Vertices satisfying $z > z_{{\text{threshold }}}$ were selected as candidates for the upper endplate. These vertices were clustered using the *pcsegdist* function, which grouped points based on a distance threshold of 1.5 mm. The largest cluster was identified as the upper endplate. To refine the region further, radial shrinkage was applied by calculating the distances of each vertex from the cluster’s centroid in the $xy$-plane:

17 $$r_{i} = \sqrt {\left( {x_{i} - C_{x} } \right)^{2} + \left( {y_{i} - C_{y} } \right)^{2} }$$

Vertices with ${r}_{i}$ greater than the 70th percentile of radial distances were excluded, creating a circularly refined upper endplate.

**Fig. 9:**
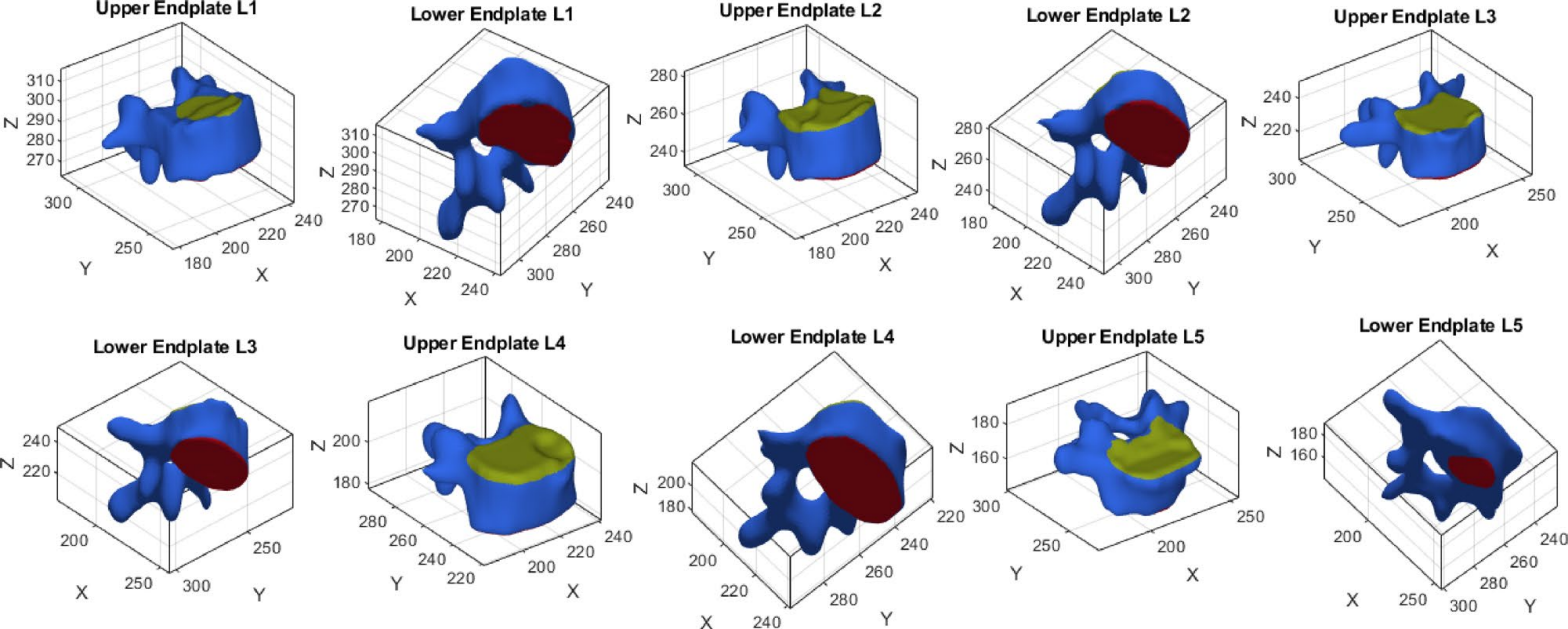
Visualization of the extracted upper (green) and lower (red) endplates for vertebrae L1 to L5. The 3D surface mesh of each vertebra is shown in blue, highlighting the precise segmentation of the endplates.

A similar approach was used for the lower endplate. A parallel plane, positioned 20 mm below the upper endplate plane, was defined. Vertices located on the opposite side of this plane were selected as candidates for the lower endplate. These points were clustered, and the largest cluster was extracted as the lower endplate. Figure 9 presents the automated segmentation of vertebral endplates from L1 to L5. The upper endplates are highlighted in green, while the lower endplates are shown in red. The surrounding vertebral structures are represented as 3D surface meshes in blue, demonstrating the precision of the segmentation process. Shrinkage was applied to this cluster by retaining only points within $55\text{\%}$ of the maximum radial distance from the cluster’s centroid.

### Modelling cartilage between each vertebra and discs

In this study, cartilage modeling between the vertebrae (L1 to L5) and the intervertebral discs was performed using the GIBBON library for generating and visualizing 3D meshes. The methodology involved extracting upper and lower endplate surfaces, identifying regions of contact between vertebrae and discs, and combining these components to create cartilage representations for FEA. The upper and lower endplates of each vertebra were first defined based on the cleaned cortical geometry and their corresponding regions using logical indexing. Next, the regions of contact between the nucleus and annulus components of the intervertebral disc and the vertebral endplates were determined. These contacting faces were extracted from the respective joint geometries of the nucleus and annulus based on predefined labels. This ensured that only the portions of the disc interacting with the vertebral endplates were included in the modeling. For example, the contact regions between the nucleus and the lower endplate of L1 were extracted, as were the annulus contact regions, following a similar procedure.

The cartilage between the vertebrae and discs was then modeled by joining the upper and lower endplate geometries with the contact regions of the nucleus and annulus. Using a dedicated function, the extracted surfaces and contact faces were combined into unified cartilage models. This process was repeated for all segments of the lumbar spine, generating cartilage geometries for each vertebra-disc interface. For example, the cartilage connecting the lower endplate of L1 to the disc below it (L1D1) was created, and similar operations were performed for all other segments.

**Fig. 10:**
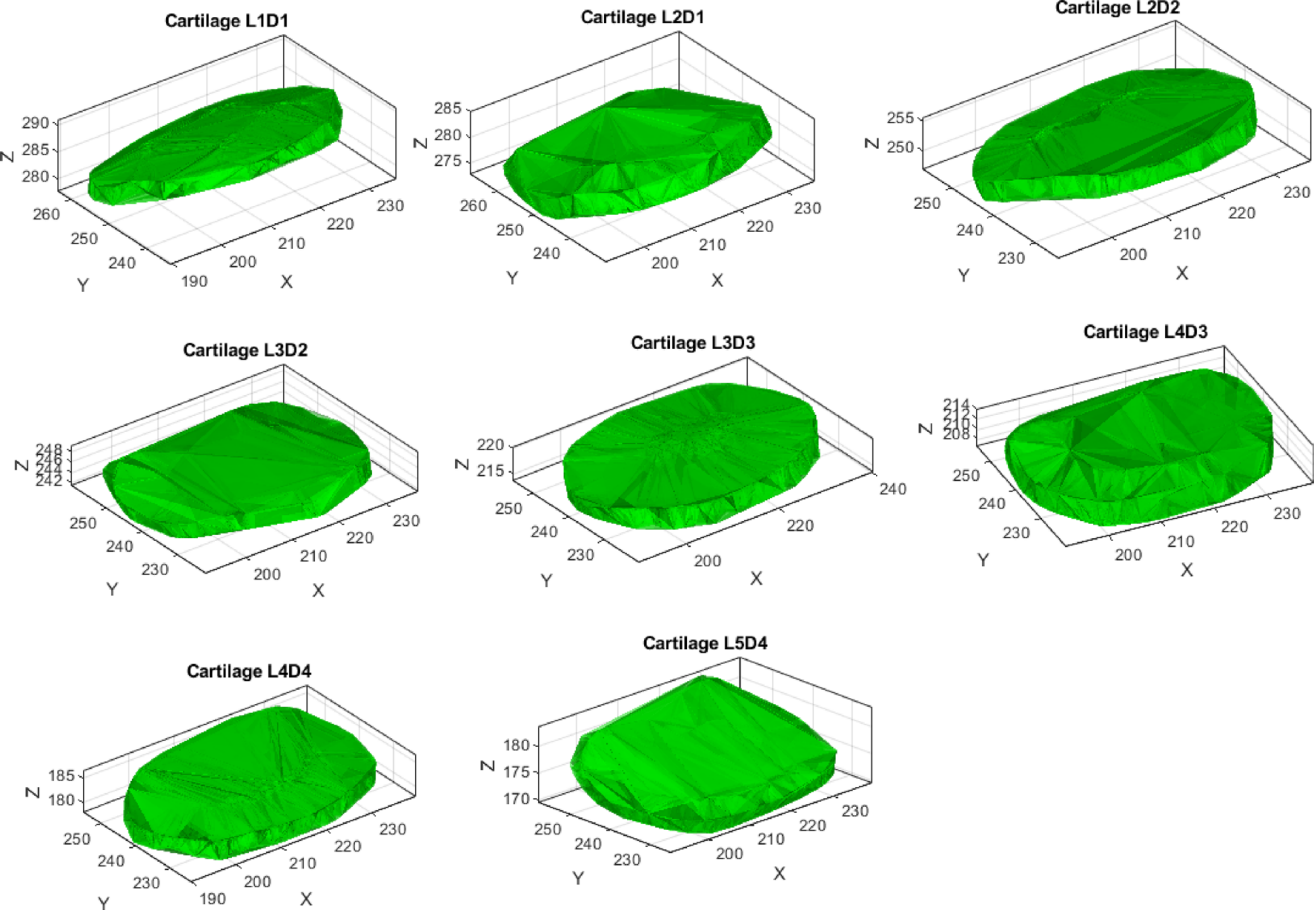
Visualization of cartilage models between vertebrae (L1 to L5) and their corresponding intervertebral discs. Each subplot represents a specific cartilage segment (e.g., L1D1, L2D1, etc.).

Figure 10 illustrates the cartilage regions modeled between vertebrae (L1 to L5) and their corresponding intervertebral discs. The labels (e.g., L1D1, L2D1, etc.) indicate the cartilage location and its association with the vertebra and disc. For instance, L1D1 represents the cartilage between vertebra L1 and the disc L1/L2, while L2D1 refers to the cartilage between vertebra L2 and the same disc (L1/L2). Similarly, L3D3 corresponds to the cartilage between vertebra L3 and the disc L3/L4. The labeling pattern continues for all vertebra-disc connections, ensuring clarity in identifying cartilage relationships across the lumbar spine.

### Finite element modeling and loading methodology

**Fig. 11:**
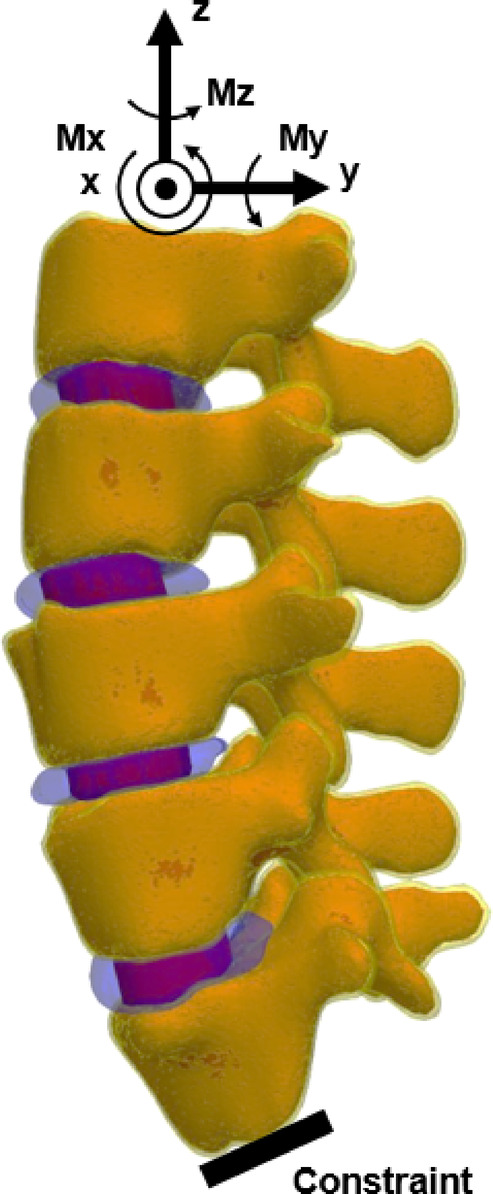
Lumbar spine finite element model illustrating the cortical and cancellous bone structures, intervertebral disc components, and cartilages between vertebrae (L1 to L5). The lower endplate of L5 is constrained, while the applied compression Load Fx, Fy, Fz and moments (Mx, My, Mz) are depicted for loading conditions.

The lumbar spine model was subjected to physiologically relevant loading scenarios, including flexion, extension, axial rotation, and lateral bending, to simulate real-world motions. Figure 11 illustrates the finite element model of the lumbar spine, encompassing vertebrae L1 to L5 along with intervertebral disc components, cartilages, cortical and cancellous bones, and the applied loading conditions.

For extension, a moment of Mx = − 7.5 Nm and a compressive force of Fz = − 50 N were applied, while flexion loading was simulated with a moment of Mx = 7.5 Nm and a compressive force of Fz = − 117.5 N. Axial rotation was analyzed under a rotational moment of Mz = 5.5 Nm and a compressive force of Fz = − 72 N. For lateral bending, the model was subjected to a moment of My = 7.8 Nm and a compressive force of Fz = − 70 N. The lower endplate of L5 was rigidly constrained with zero degrees of freedom for translation or rotation to provide stability. Loading was applied incrementally using a quasi-static approach to ensure numerical stability.

Table 2 summarizes the material properties and behavioral characteristics assigned to each component of the lumbar spine model. Cortical and cancellous bones are modeled as isotropic elastic materials, with the cortical bone exhibiting a Young’s modulus of 12,000 MPa and Poisson’s ratio of 0.3, highlighting its higher stiffness and load-bearing capacity. The cancellous bone, with a Young’s modulus of 100 MPa and Poisson’s ratio of 0.2, represents the internal spongy structure with lower stiffness. The nucleus and annulus, forming the intervertebral discs, are modeled using the Mooney–Rivlin material model to accurately capture their hyperelastic deformation behavior. The nucleus has Mooney–Rivlin parameters c_1_ = 0.12, c_2_ = 0.09, and a bulk modulus of 666.67 MPa, while the annulus is characterized by c_1_ = 0.56, c_2_ = 0.14, and a bulk modulus of 14.89 MPa. Both components have a density of 1.0003 g/cm^3^. Cartilage is treated as an isotropic elastic material with a Young’s modulus of 23.8 MPa, Poisson’s ratio of 0.4, and a density of 1.0003 g/cm^3^, facilitating smooth load transfer between vertebrae and discs. Ligaments (ALL, PLL, ITL, LF, CL, ISL-SSL) are modeled with a Young’s modulus of 0.22 MPa and represented using 10 spring elements per ligament to simulate their biomechanical response. The upper endplate of L1 and the lower endplate of L5 are defined as rigid bodies to ensure structural constraints at the boundaries. These material properties, including hyperelastic coefficients for the nucleus and annulus, were validated through sensitivity analysis to ensure the robustness and predictive accuracy of the finite element model. Contact boundary conditions were defined between interacting components to simulate physiological constraints. Stick contact was implemented between the nucleus and annulus to allow relative motion while ensuring continuity. The cartilage-endplate interface was modeled using tied contact to prevent penetration or gaps during deformation. Ligament attachment areas were constrained using node-to-surface contact algorithms to represent anatomical connections. A tolerance of 0.01 mm was applied to prevent overlapping nodes at boundaries, enhancing numerical stability.

**Table 2:** Summary of the lumbar spine model components, including the number of volumetric elements, surface elements, and vertices for cortical and cancellous bones, nucleus, annulus, and cartilage across different regions (L1 to L5).

Component	Region	Volumetric elements	Surface elements	Vertices
Cortical bone	L1	276,374	44,988	65,517
	L2	282,049	45,512	66,887
	L3	294,735	48,282	70,171
	L4	256,790	42,210	61,369
	L5	275,650	45,962	65,963
Cancellous bone	L1	76,122	11,270	15,760
	L2	79,964	11,020	16,331
	L3	90,909	12,146	18,462
	L4	83,500	10,386	16,745
	L5	83,338	11,630	17,046
Nucleus	D1(L1/L2)	4170	936	956
	D2(L2/L3)	4300	960	983
	D3(L3/L4)	4218	986	978
	D4(L4/L5)	5829	1136	1285
Annulus	D1(L1/L2)	4111	1494	1114
	D2(L2/L3)	4396	1612	1197
	D3(L3/L4)	4160	1606	1159
	D4(L4/L5)	5550	1842	1448
Cartilage	L1D1	15,765	31,530	2731
	L2D1	19,067	38,134	3351
	L2D2	17,130	34,260	3083
	L3D2	18,144	36,288	3299
	L3D3	21,001	42,002	3678
	L4D3	19,062	38,124	3394
	L4D4	18,280	36,560	3215
	L5D4	18,261	36,522	3158
Total		1,982,875	587,398	449,280

The nucleus pulposus was modeled with no gaps at the vertebral endplates, ensuring continuous load transfer. Wrapping was accounted for by aligning the nucleus geometry with the curvature of the endplates using a shrinkage factor derived from anatomical data. The annulus fibrosus was connected to the nucleus through shared nodes at the boundaries, ensuring seamless integration and reducing stress singularities. The gap between the nucleus pulposus and annulus fibrosus was minimized to maintain physiological consistency. Additionally, the cartilages were modeled using the vertebral endplates as reference geometries, with the gaps between the endplates and the intervertebral discs fully filled with cartilage material. This approach ensured accurate load transfer and anatomical fidelity, reinforcing the biomechanical integrity of the finite element model. Table 2 provides information about the mesh characteristics used in the FEA. The cortical bone, due to its thickness and structural complexity, requires a finer mesh, resulting in the highest number of volumetric elements.

In contrast, the cancellous bone, being more porous and less structurally demanding, is represented with a coarser mesh, leading to fewer volumetric elements. The intervertebral discs, including the nucleus and annulus, are modeled with smaller volumetric element counts to focus on their localized deformation behavior. Cartilage regions, situated between vertebrae and discs, are finely meshed to ensure representation of their role in load transfer and articulation. The table also highlights the surface element counts and vertex numbers for each component, demonstrating the overall resolution and quality of the mesh. The total volumetric element counts of 1,982,875 and the surface element count of 587,398 emphasize the complexity and precision of the model, ensuring reliable simulation results under various loading conditions.

### Extension loading analysis

In this study, we implemented an automated FEA pipeline to evaluate the biomechanical response of the lumbar spine under extension loading conditions. The workflow included deep learning-based segmentation (nnUNet), automated meshing (Gibbon library), and high-fidelity simulation using FEBio. The model accurately differentiates cortical bone, cancellous bone, intervertebral discs, and ligamentous structures, ensuring precise representation of anatomical and mechanical properties. For the extension scenario, the lumbar spine was subjected to a moment of 7.5 Nm and a compressive force of 50 N along the Fz-axis to simulate backward bending. Additional boundary conditions included Fx, Fy, and Mx, My, Mz moments applied to mimic physiological constraints. The L5 lower endplate was fully constrained, representing a fixed boundary condition commonly used in spinal biomechanics studies.

**Fig. 12:**
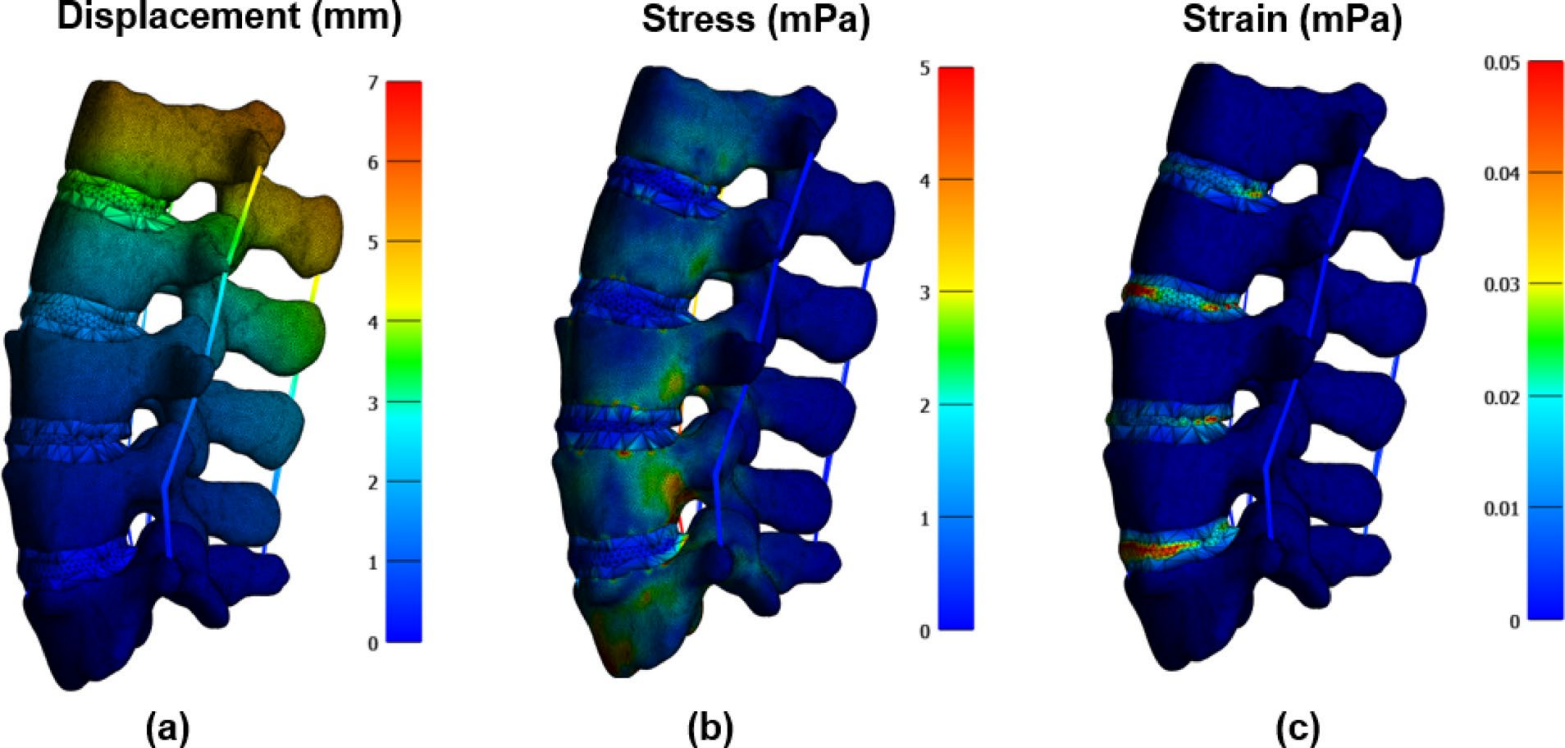
FEA of the lumbar spine under extension loading. (**a**) Displacement contours highlight greatest deformation at the superior vertebrae and least at the constrained L5. (**b**) Stress distribution reveals peak values in the posterior elements, especially pedicles and facet joints. (**c**) The strain distribution (in MPa) highlights areas of high deformation in the posterior ligaments and the posterior annulus fibrosus, indicating their critical role in limiting the range of motion during extension. Color scales indicate the magnitude of the respective variables, with red representing peak values and blue representing the lowest.

The simulation results indicate that extension forces induce posterior compression and anterior tension across the vertebral column^23^. As shown in Fig. 12, the stress and strain distributions reveal elevated forces in the posterior elements, including the facet joints, pedicles, and laminae, which play a crucial role in stabilizing the spine. The intervertebral discs, particularly the annulus fibrosus, experienced tensile and shear stresses in the posterior regions due to nucleus pulposus displacement. This aligns with clinical observations where posterior disc herniations and spondylolysis (pars interarticularis stress fractures) are frequently linked to repetitive extension movements. The displacement contours illustrate greater deformation in the anterior vertebral regions, highlighting the spinal flexibility required for extension movements. However, excessive extension forces can lead to microdamage in osteoporotic patients, increased facet joint loading, and accelerated degenerative changes in individuals with pre-existing spinal conditions.

**Fig. 13:**
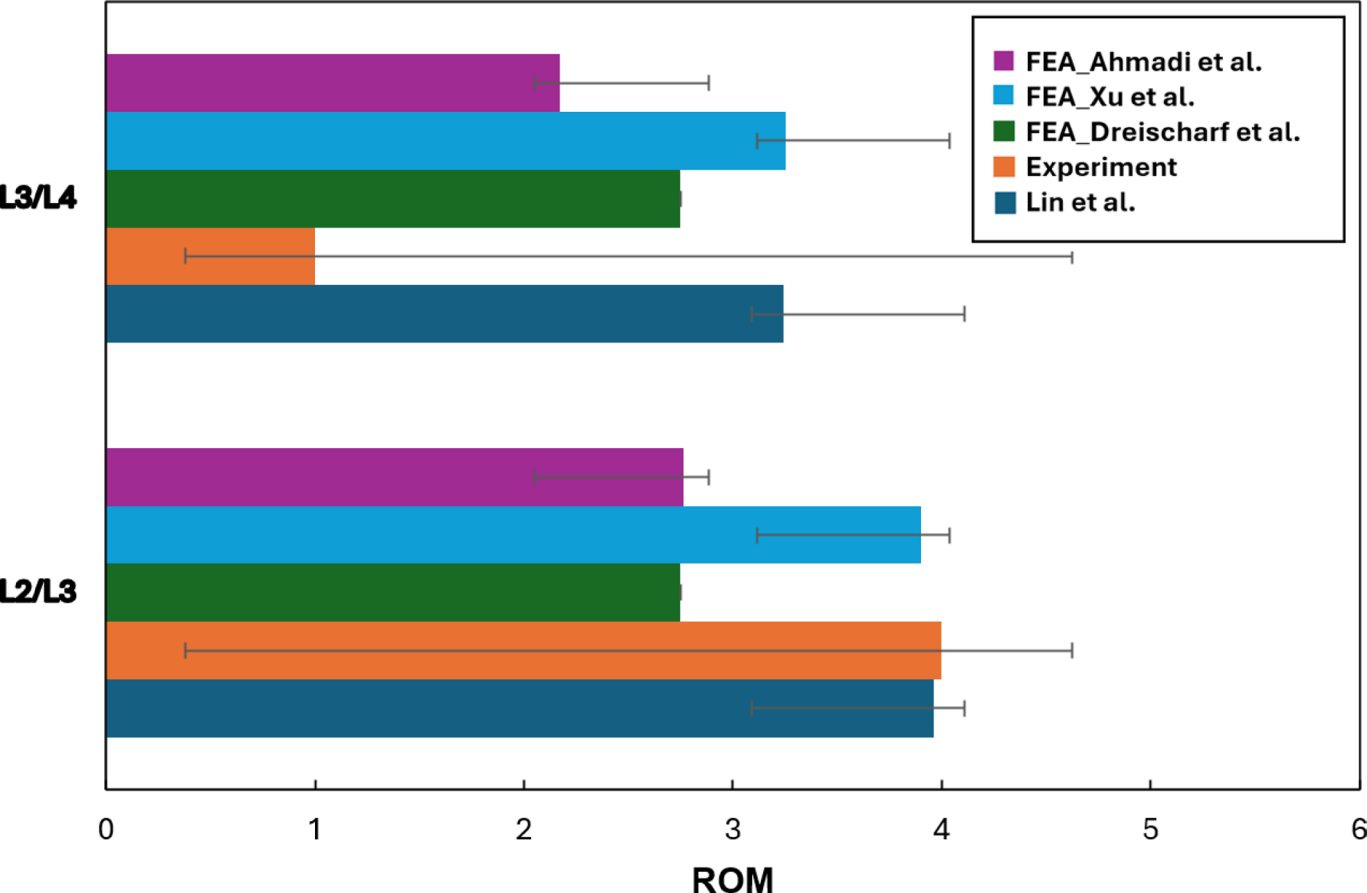
Comparison of ROM at L2/L3 and L3/L4 levels between the FEA studies )Ahmadi et al.^23^, Xu et al.^27^ and Dreischarf et al.^28^), experimental results^29^, and Lin et al.^30^. Error bars represent variability in the reported data.

Compared to traditional manual segmentation and meshing, our automated approach significantly reduces processing time, improves reproducibility, and minimizes user-induced variability. By integrating deep learning segmentation with physics-based simulations, the method provides an efficient and clinically relevant framework for studying spinal biomechanics. The validated FEA model has potential applications in surgical planning, implant design, and rehabilitation strategies, particularly for evaluating spinal fixation devices and fusion techniques under extension loading conditions. Figure 13 presents a comparative analysis of the ROM at L2/L3 and L3/L4 levels under extension loading conditions^23^. The results presented in this study are based on our previous work^23^ and are compared with other FEA studies (Xu et al.^27^, Dreischarf et al.^28^), experimental data from Pearcy^29^, and findings from Lin et al.^30^.

This comparison evaluates the reliability and accuracy of the developed model in predicting lumbar spine kinematics. The ROM values in the present study align closely with experimental results and previous computational models, demonstrating the effectiveness of our automated segmentation and meshing pipeline in capturing lumbar spine motion dynamics. The error bars indicate the variability in reported data, reflecting the differences in methodologies, material properties, and boundary conditions across studies. At the L2/L3 level, the present model predicts a ROM slightly lower than Xu et al. but closely matches the experimental findings. In contrast, at the L3/L4 level, the ROM prediction of our model is within the range reported by Lin et al., indicating a consistent response under extension loading. The variations observed in ROM predictions across different studies may be attributed to differences in ligament modeling, material properties, and contact definitions. The close agreement between our automated FEA model and experimental results supports its validity for biomechanical analysis, with potential applications in clinical diagnostics, implant evaluation, and surgical planning for spinal disorders.

The current study demonstrates the successful application of our automated pipeline to the complete lumbar spine of a single subject, encompassing all five vertebral segments from L1 to L5. The modular design of the pipeline, which incorporates parametrically defined geometric transformations and ligament attachments, enables significant adaptability to different anatomical variations. For each new subject, users can adjust key parameters to optimize the fit for unique geometries, a process that remains substantially faster and more reproducible than traditional manual modeling.The robustness of our approach is further enhanced by utilizing a pre-trained deep learning model for segmentation, which was trained on a diverse dataset that includes a wide range of anatomical variations and pathologies. Although this study does not yet present results for pathological or post-surgical spines, the adaptability of our parametric approach establishes a strong foundation for future research in these areas. Subsequent studies will focus on validating the pipeline’s robustness and accuracy across cohorts of subjects with various pathologies, including scoliosis and degenerative disc disease, to fully demonstrate its clinical potential.

While several recent studies have proposed partially automated or deep learning-driven FEA pipelines, our work is distinguished by delivering a fully automated, end-to-end framework that extends from image segmentation to model assembly. In contrast, many existing pipelines, even those utilizing deep learning, still require significant manual intervention in stages such as meshing, ligament attachment, or the separation of anatomical components (e.g., nucleus and annulus). This reliance on manual steps introduces user variability and increases the time required for model generation. Our method overcomes this bottleneck by eliminating manual intervention. In terms of quantitative performance, the speed of our pipeline is a notable advantage, reducing model generation time from days or weeks to just hours a substantial improvement over both manual and semi-automated approaches. Furthermore, the accuracy of our models is evidenced by the close alignment of our predicted ROM values with both experimental data and values reported in the literature, matching the performance of other high-quality FEA models. The robustness of our pipeline, achieved using a pre-trained deep learning model on a diverse dataset, makes it especially well-suited for handling anatomical variations among subjects.

## Discussion

This study presents a novel automated technique for biomechanical analysis of the lumbar spine, integrating state-of-the-art deep learning algorithms for segmentation with advanced finite element modeling. Traditional methods often require manual segmentation and meshing, which introduce variability and significantly increase processing time. In contrast, our approach leverages the GIBBON library and FEBio to automate these steps, resulting in substantial improvements in both accuracy and efficiency. The proposed pipeline ensures high-fidelity representations of complex anatomical structures including cortical and cancellous bone, ligaments, cartilage, and intervertebral discs. By minimizing manual intervention, this technique enhances reproducibility and enables more reliable simulations of spinal biomechanics under various loading conditions. Beyond its computational advantages, this method offers significant clinical implications. Automation of lumbar spine modeling enables rapid, patient-specific simulations, which can be instrumental in preoperative planning and surgical decision-making. Personalized models generated through our pipeline allow for the anticipation of mechanical complications, optimization of implant designs, and reduction of postoperative risks. Furthermore, the ability to efficiently produce detailed and precise biomechanical models reduces the costs associated with traditional manual modeling and lengthy simulations. This improved accessibility may facilitate early diagnosis and intervention, potentially reducing the long-term healthcare burden of degenerative spinal disorders. The enhanced precision in biomechanical simulations presents a promising avenue for improving diagnostic accuracy and treatment strategies, ultimately contributing to better patient outcomes.

The choice of element type is a critical consideration in finite element modeling, particularly in biomechanics. While our current automated pipeline efficiently generates tetrahedral meshes, we acknowledge the preference for hexahedral elements in many biomechanical studies due to their superior performance in capturing bending and shear deformation and their ability to reduce volumetric locking. The automation of tetrahedral mesh generation, as implemented in this study, provides a robust solution for handling the complex geometries derived directly from patient CT scans—a task that remains challenging and time-consuming for hexahedral meshing, even with commercial software. Looking ahead, a key direction for our future work is to extend our automated framework to support the hexahedral mesh generation. This would involve developing new algorithms that utilize the anatomically accurate segmentation from our current pipeline to create a structured grid or adopt a hybrid approach that combines automated meshing techniques with our segmentation workflow. Such advancements would further enhance the accuracy and computational efficiency of patient-specific spine modeling, provide the benefits of a fully automated workflow while meet the biomechanical requirements of high-quality hexahedral meshes.

Validation of finite element models against experimental data is essential for establishing physiological accuracy. While the primary focus of this study is the introduction and demonstration of a novel automated pipeline for model generation, we have performed rigorous benchmarking analyses to ensure our model outputs are consistent with established biomechanical data. As presented in our results, the predicted ROM under extension loading was compared with both previously published numerical results and experimental cadaveric data from the literature. The close alignment between our model’s predictions and these reference studies provides confidence in the anatomical and physiological accuracy of our methodology. Additionally, a key contribution of our pipeline is its ability to generate high-fidelity representations of intricate components, such as the distinct geometries of cortical/cancellous bone, annulus fibrosus, and nucleus pulposus, which cannot be directly extracted from standard CT or MRI images. This mathematical and computational approach ensures that the model’s internal structures are represented with a high degree of anatomical fidelity. We acknowledge, however, that a more comprehensive validation using our own cadaveric data is an essential next step. Future work will involve subjecting pipeline-generated models to a full range of experimental tests, such as validating intradiscal pressures and facet forces, further solidifying the clinical applicability of our methodology.

A major advantage of our automated pipeline is its efficiency relative to conventional methods. While we qualitatively describe the substantial reduction in manual intervention, we recognize that the manuscript lacks explicit quantitative benchmarks for total computational runtime. Nevertheless, the primary benefit of our methodology is the transformation of a multi-day, labor-intensive manual process into a workflow requiring only minutes of user interaction. Total computational runtime including segmentation, meshing, ligament generation, and FEA simulation depends on hardware specifications and CT data complexity. In future work, we plan to conduct a detailed quantitative analysis of computational runtimes across different hardware configurations, providing explicit benchmarks that compare our automated pipeline with semi-automated and manual workflows to further substantiate the efficiency claims of this study. The development of an automated ligament modeling approach is another significant advance toward a fully automated FEA pipeline. The spherical coordinate-based method described here efficiently and consistently defines ligament attachment points without manual intervention. The parameters used in this method, as detailed in Table 1, were carefully selected based on a comprehensive review of anatomical atlases and biomechanical literature to ensure plausible ligament paths and orientations. While this method is highly reproducible, quantitative validation of its anatomical accuracy remains essential. A key focus for future work will be benchmarking the ligament paths and orientations generated by our automated pipeline against ground truth data from high-resolution imaging (e.g., MRI-based segmentations of cadaveric specimens) or detailed manual segmentation using anatomical atlases. This will provide a quantitative assessment of our method’s anatomical fidelity and further refine the coordinate parameters to improve physiological accuracy.

A direct comparison between the outputs of our automated pipeline and those from manually generated models is a critical and highly relevant benchmark. While the primary objective of this study is to demonstrate the significant reduction in time and manual effort during pre-processing a process that can take a trained engineer days or weeks—we recognize the importance of validating the final FEA results against such a gold standard. In this manuscript, we have validated our model’s Range of Motion against established experimental and numerical literature, demonstrating physiologically consistent results. In future work, we propose a dedicated study in which an expert-generated manual model of the same subject is created for direct quantitative comparison. Such a study would enable a detailed analysis of differences in RoM and stress distribution, as well as a comprehensive comparison of mesh quality metrics and, crucially, the total time required for model creation. This approach will provide definitive evidence of the efficiency and accuracy of our proposed automated framework.

### Future work

Future development of this automated technique may include dynamic modeling capabilities, enabling the simulation of spinal motion with greater physiological accuracy by incorporating muscle forces and more complex loading scenarios. The scalability of our method extends beyond the lumbar spine, offering potential applications in other regions of the spine and various orthopedic joints. By broadening the use of this approach, the proposed automated framework could transform computational biomechanics, providing more comprehensive insights into musculoskeletal health and informing orthopedic treatment strategies.

While the automation of multiple pre-processing stages from segmentation to meshing greatly enhances efficiency, it also introduces the potential for small errors to propagate through the workflow. Although the core objective of this study is to demonstrate the feasibility and efficiency of a fully automated model generation pipeline, we recognize the importance of understanding the sensitivity of FEA results to upstream parameters. Our current methodology addresses these risks by employing a robust, pre-trained deep learning model for segmentation and mesh refinement techniques designed to preserve anatomical fidelity. Nonetheless, conducting a formal sensitivity analysis—systematically investigating the effects of segmentation tolerances and mesh density on key FEA outputs such as ROM and peak stress values—is an essential area for future research. Such analysis would provide a deeper understanding of the model’s robustness and inform the selection of optimal parameters for balancing computational cost with predictive accuracy.

The accurate modeling of spinal ligaments is another crucial aspect of biomechanically realistic FEA models. While ligaments exhibit complex, nonlinear, and viscoelastic stress–strain behavior including a characteristic toe-region—our current pipeline employs a simplified linear spring element model with fixed stiffness values. This design choice was made to achieve a computationally efficient and robust platform for demonstrating the feasibility of a fully automated pre-processing workflow. We acknowledge that this simplification does not capture the full physiological response of the ligaments. Moving forward, the modular nature of our pipeline will allow for the integration of advanced, non-linear constitutive models that better represent the toe-region and viscoelastic properties of ligaments. This enhancement will be an important next step toward improving the physiological fidelity of our simulations and will enable more comprehensive analyses of spinal biomechanics under diverse loading conditions.

### Limitation

While our automated pipeline is designed to be robust and highly reproducible, we acknowledge that, like any automated system, it has certain limitations. The primary areas where manual intervention may be necessary include cases with severely degraded image quality, which can challenge even the most robust deep learning segmentation models. Furthermore, while the pipeline is designed to be adaptable to anatomical variations, extreme pathologies, such as severe scoliosis, large osteophytes, or the presence of extensive post-surgical hardware, may require user-specific adjustments to the parametric settings for geometric extraction and ligament attachment. The pipeline’s modularity, however, is a key strength in these scenarios, as it allows for targeted manual corrections without necessitating a complete restart of the entire workflow. For instance, a user could manually refine the segmentation of a fused vertebra and then re-run the meshing and ligament attachment steps, still achieving a significant reduction in overall processing time compared to a fully manual approach. A comprehensive evaluation of the pipeline’s performance across a wide spectrum of pathologies will be the focus of our future work, which will further quantify the robustness and limitations of our approach. While our automated pipeline is designed to be robust and highly reproducible, we recognize that, like any automated system, it does have certain limitations. Manual intervention may still be necessary in specific scenarios, particularly when confronted with severely degraded image quality, which can pose significant challenges even for advanced deep learning segmentation models. Additionally, although the pipeline is engineered for adaptability to a wide range of anatomical variations, extreme pathologies such as severe scoliosis, prominent osteophytes, or extensive post-surgical hardware may require user-specific adjustments to parametric settings during geometric extraction or ligament attachment.

The modular structure of the pipeline is a key strength in such cases, enabling targeted manual corrections without necessitating a complete restart of the workflow. For example, if segmentation of a fused vertebra requires manual refinement, the user can perform this adjustment and subsequently re-run only the meshing and ligament attachment steps, still achieving a substantial reduction in overall processing time compared to fully manual approaches.A evaluation of the pipeline’s performance across a broad spectrum of pathologies will be a primary focus of future work. This will enable us to further quantify both the robustness and the limitations of our approach.

## Conclusion

The automated method developed in this study streamlines the traditionally complex process of biomechanical model generation and simulation for the lumbar spine. By integrating deep learning-based segmentation with an automated finite element modeling workflow, our approach minimizes manual intervention while preserving anatomical accuracy. The methodology enables the precise extraction and meshing of cortical and cancellous bone, intervertebral discs, ligaments, and cartilage, ensuring that generated models closely resemble physiological structures. This pipeline facilitates rapid and reproducible analyses, which are essential for both research and clinical applications. The application of this method to simulate extension loading conditions highlights its ability to capture the lumbar spine’s biomechanical response with high fidelity. Our results show that applying an extension moment of − 7.5 Nm and a compressive force of − 50 N produces stress concentrations at the posterior spinal elements, particularly at the facet joints and pedicles. These regions bear the highest compressive loads, while the anterior vertebral bodies are subjected to tensile forces. The intervertebral discs exhibit posterior-directed nucleus displacement, consistent with clinical observations of disc herniation patterns. Additionally, the annulus fibrosus demonstrates increased tensile stress, especially in its posterior regions, reflecting its critical role in spinal stability. These findings underscore the importance of understanding extension-induced stress patterns, as excessive or repetitive loading may contribute to degenerative conditions such as spondylolysis.

The validation presented in this study is further supported by our previously published work, which provides a comprehensive experimental and computational analysis of lumbar spine biomechanics under similar loading conditions^23^. The consistency between these studies highlights the reliability of our automated modeling approach and its effectiveness in reproducing physiologically relevant biomechanical responses. Further validation of the automated modeling pipeline is provided by the close alignment of our predicted ROM values at L2/L3 and L3/L4 with experimental data and previous computational studies. This demonstrates the method’s reliability in capturing lumbar spine kinematics. The consistency between the present study’s predictions and established FEA models indicates that automated segmentation and meshing do not compromise simulation accuracy. Nevertheless, observed variability across different models highlights the influence of factors such as ligament representation, material properties, and contact conditions on ROM outcomes, underscoring the need for careful parameter selection in future studies. Beyond modeling efficiency, this automated approach has significant clinical implications. The ability to generate patient-specific finite element models with minimal manual effort enhances preoperative planning, allowing surgeons to assess spinal stability and predict the effects of surgical interventions. Automation of segmentation and meshing reduces user-dependent variability and ensures reproducible results across cases. This framework could be extended to study other spinal conditions, such as degenerative disc disease, scoliosis, and trauma-induced injuries, broadening its applicability in orthopedic research.

Looking forward, future developments could focus on incorporating dynamic simulations to assess real-time biomechanical responses under various loading scenarios. Further automation of material property assignment based on patient-specific imaging data could improve model accuracy. Additionally, integrating muscle forces and soft tissue mechanics into the simulation framework would offer a more comprehensive representation of spinal biomechanics. Expanding the method to other spinal regions and musculoskeletal structures could extend its utility to a broader range of biomechanical investigations. This study demonstrates that automated segmentation and finite element modeling can yield anatomically accurate and biomechanically relevant spinal models, providing a scalable, reproducible, and efficient approach to spinal analysis. With continued refinement, this methodology has the potential to enhance both research and clinical practice, improving diagnostics, treatment planning, and implant design in spinal biomechanics.

The clinical relevance of our automated pipeline is directly linked to its ability to rapidly generate patient-specific finite element models. While this study primarily focuses on the technical development and benchmarking of the pre-processing methodology, the resulting models are immediately applicable to a variety of clinical scenarios. For instance, in preoperative planning, a surgeon can utilize our pipeline to generate a biomechanically accurate model from a patient’s CT scan in just a few hours, rather than the weeks required by conventional approaches. These models can then be used to virtually simulate different surgical interventions, such as the placement of lumbar interbody fusion cages or pedicle screw fixation. By analyzing the stress, strain, and stability of these constructs, surgeons can evaluate and optimize implant selection and placement prior to surgery. This capability for rapid, patient-specific simulation offers a powerful tool for anticipating complications, refining surgical strategies, and ultimately improving patient outcomes. The transition from this lab prototype to a fully integrated clinical tool, however, will involve several key stages. Firsexportftware must be packaged as a DICOM-compliant module to enable seamless integration with hospital information systems such as PACS, facilitating automated ingestion of patient CT data and export of clinically relevant outputs including interactive 3D models and biomechanical analyses to surgical planning software. Second, regulatory approval from agencies such as the U.S. Food and Drug Administration (FDA) and CE Mark certification will be required, necessitating extensive validation across large patient cohorts and rigorous documentation of the software development lifecycle. Finally, ensuring high surgeon acceptance is critical; this will require an intuitive user interface, demonstration of clinical benefits through pilot studies, and comprehensive training for end users. The foundational technology established in this work paves the way for these future developments, enabling faster, more accurate, and scalable biomechanical analyses in clinical contexts.

## Data Availability

The code supporting this study is publicly available at Zenodo: 10.5281/zenodo.17051686.
